# Racial Discrimination, Religious Coping, and Cardiovascular Disease Risk Among African American Women and Men

**DOI:** 10.1007/s40615-024-02113-x

**Published:** 2024-08-19

**Authors:** Jason Ashe, Keisha Bentley-Edwards, Antonius Skipper, Adolfo Cuevas, Christian Maino Vieytes, Kristie Bah, Michele K. Evans, Alan B. Zonderman, Shari R. Waldstein

**Affiliations:** 1 Laboratory of Epidemiology and Population Sciences, National Institute On Aging, Baltimore, MD USA; 2 Duke Global Health Institute, Duke University, Durham, NC USA; 3 Duke Cancer Institute, Duke University, Durham, NC USA; 4 Samuel DuBois Cook Center On Social Equity, Duke University, Durham, NC USA; 5 The Gerontology Institute, Georgia State University, Atlanta, GA USA; 6 Department of Social and Behavioral Sciences, New York University School of Global Public Health, New York, NY USA; 7 Center for Anti-Racism, Social Justice, and Public Health, New York University School of Global Public Health, New York, NY USA; 8 Department of Psychology, University of Maryland, Baltimore County, Baltimore, MD USA; 9 Division of Gerontology, Geriatrics, and Palliative Medicine, University of Maryland School of Medicine, Baltimore, MD USA

**Keywords:** Racial discrimination, Religious coping, Cardiovascular disease risk, African Americans

## Abstract

**Objective:**

This cross-sectional study examined whether religious coping buffered the associations between racial discrimination and several modifiable cardiovascular disease (CVD) risk factors—systolic and diastolic blood pressure (BP), glycated hemoglobin (HbA1c), body mass index (BMI), and cholesterol—in a sample of African American women and men.

**Methods:**

Participant data were taken from the Healthy Aging in Neighborhoods of Diversity Across the Life Span study (*N* = 815; 55.2% women; 30–64 years old). Racial discrimination and religious coping were self-reported. CVD risk factors were clinically assessed.

**Results:**

In sex-stratified hierarchical regression analyses adjusted for age, socioeconomic status, and medication use, findings revealed several significant interactive associations and opposite effects by sex. Among men who experienced racial discrimination, religious coping was negatively related to systolic BP and HbA1c. However, in men reporting no prior discrimination, religious coping was positively related to most risk factors. Among women who had experienced racial discrimination, greater religious coping was associated with higher HbA1c and BMI. The lowest levels of CVD risk were observed among women who seldom used religious coping but experienced discrimination.

**Conclusion:**

Religious coping might mitigate the effects of racial discrimination on CVD risk for African American men but not women. Additional work is needed to understand whether reinforcing these coping strategies only benefits those who have experienced discrimination. It is also possible that religion may not buffer the effects of other psychosocial stressors linked with elevated CVD risk.

**Supplementary Information:**

The online version contains supplementary material available at 10.1007/s40615-024-02113-x.

## Introduction

Racial discrimination has been largely implicated in racial health disparities across various cardiovascular diseases (CVDs) and related risk factors [1, 2, 3, 4]. African American adults carry a disproportionate burden of CVD risk factors like obesity and hypertension and experience earlier onset and greater mortality risk due to CVDs [5, 6, 7, 8]. Equally, compared to all other racial and ethnic groups in the USA, African American adults report more exposure and vulnerability to racial discrimination across commonplace settings [9, 10, 11, 12, 13]. Racial discrimination is an established chronic stressor seemingly leading to detrimental health consequences, acting upon multiple psychological, biobehavioral, and physiological pathways (e.g., poorer emotional regulation, greater depressive symptoms, engagement in maladaptive coping strategies, low-grade inflammation, and cardiac reactivity [14, 15, 16].Research has documented associations between experienced racial discrimination and elevated blood pressure (BP), higher body mass index (BMI), and worse cardiometabolic health [17, 18, 19, 20, 21, 22, 23]. It remains unclear if these effects are more striking in African American women versus men [24, 25]. Nevertheless, discrimination exerts a cumulative impact on their overall health and well-being and is a fundamental contributor to racial disparities across cardiovascular diseases. Efforts to mitigate these racial health disparities must also identify protective factors that can counteract discrimination’s health impacts.

Markedly, a large body of literature has shown promising associations between frequent religious participation and better cardiovascular health as well as decreased mortality risk among African American adults [26, 27, 28, 29, 30, 31, 32]. Compared to other racial and ethnic groups in the USA, African American adults (and women more so than men) exhibit the highest levels of religiosity across multiple indicators of engagement (e.g., religious service attendance, religious coping use, prayer) [33, 34, 35, 36, 37, 38]. Religion is intimately connected to their fortitude and survival through centuries of longstanding oppression and racial discrimination [39]. Expressly, in the face of relentless racism, the institutional black church has been a cornerstone of support to combat social inequity [39, 40, 41, 42]. Liturgical foci in predominantly black churches regularly integrate racial empowerment, hope, and themes of resilience into sermonic teachings and corporate worship [40, 43, 44]. Fittingly, religious African American adults commonly turn to prayer and church-based social support as coping strategies to deal with racial discrimination, too [45].

Religious coping practices have been shown to yield health benefits, even for those who experience chronic stress and unfair treatment [46, 47, 48, 49, 50, 51]. Select coping strategies have been shown to “buffer” (or reduce) the health detriments associated with experiencing chronic stress [52, 53, 54]. It is theorized that coping strategies like prayer and meditation can help inhibit stress-related physiological pathways by diminishing cardiac reactivity as well as inflammatory and neuroendocrine responses [55, 56, 57, 58, 59, 60, 61]. Religious people are also less likely to engage in risky lifestyle behaviors (e.g., binge drinking), display fewer depressive symptoms, and repeatedly rely on their church-based social support networks for help, which in turn have positive downstream effects on their overall health [62, 63, 64, 65]. Few studies have explored the potential buffering effects of religious coping on the relationships between discrimination and CVD risk factors, but findings have been inconclusive [66]. Correspondingly, if these potential buffering influences do exist, there are at least two critical reasons why these associations might vary by sex.

First, African American women and men experience and self-report racial discrimination differently. African American women, who live at the interstices of being both black and female, are often invisibilized in the discourse of racism, despite facing mistreatment, sexist and racial epithets, microaggressions at school or on their jobs, discrimination from law enforcement, and numerous other circumstances where they are disrespected or undervalued [13, 67, 68, 69, 70, 71, 72, 73, 74]. However, African American men often self-report more experienced race-based discrimination compared to women [9, 13, 75]. One reason for this might be that stereotypical depictions of black men regularly portray them as threatening and violent, which contributes to more frequent, hostile interactions with law enforcement, colleagues at work, and professionals in academic settings [76, 77, 78]. By and large, though, for men, these experiences with racial discrimination have been frequently linked to heightened CVD risk [79, 80]. In this way, the detrimental effects of these relationships may be exacerbated for African American men because of their salience to race-related discrimination.

Second, with respect to religious participation and associated coping use, there are noticeable sex differences. Women are more religious than men, and they are more likely to turn to their religious communities to make sense of and cope with stressful experiences [35, 65, 81, 82]. However, studies have found mixed results regarding religiosity and CVD risk, with some observing poorer outcomes among religious African American women and men when compared to their less religious counterparts [29, 83, 84, 85, 86, 87]. As African American men are typically less religious than their female counterparts, it is possible that, in the face of racial discrimination, they perceive religious coping as a unique source of comfort. When traumatic life events (racial discrimination, family conflict, unemployment) occur, African American men who turn to religious coping may do so because the situations seem beyond their control, especially because they are far less likely to seek support from friends, health care professionals, or counseling services than women [88, 89, 90]. Consequently, if men turn to religion as a potential stress-buffering resource in the context of racism and discrimination, these moderating effects might be more prominent for them than for women, who, despite their routine religiosity, still turn to religion for comfort but do so irrespective of the type of stress they face [91].

To our knowledge, no study has examined the interactive effects of religious coping on the associations between racial discrimination and CVD risk explicitly among African American men and women. This study used cross-sectional data from the Healthy Aging in Neighborhoods of Diversity Across the Life Span (HANDLS) study to address this inquiry. We included several modifiable CVD risk factors (systolic and diastolic BP, BMI, fasting glycated hemoglobin (HbA1c), and total cholesterol) commonly screened in primary care settings, as prior research tends to rely heavily on BP and hypertensive status [92, 93]. Moreover, we only focused on African American adults, given the sociohistorical and cultural backdrops of racism and black American religion. We seek to provide additional insight into the role of religion as a protective and resilience factor that explains the heterogeneity across health outcomes among African American adults who experience race-related stress. Lastly, several mechanisms through which religious coping use might diminish the biological effects of racial discrimination on CVD risk (better emotional and psychological wellbeing, healthier lifestyle behaviors, an increased social support network, and better stress-physiological regulation) are plausible [46, 49, 94, 95, 96, 97, 98, 99]. Thus, we conducted sensitivity testing to determine if these relationships withstood adjustment for additional psychological, biobehavioral, social, and biomedical covariates. We hypothesized that the lowest levels of CVD risk factors would be observed among African American men who experienced racial discrimination but also frequently used religion to cope. For women, we suspected potential buffering effects would still emerge but would be less striking than for men.

## Methods

### *Sample and Participants*

The HANDLS is an ongoing longitudinal cohort study that examines health disparities attributable to race and socioeconomic status (SES). The HANDLS comprises a fixed cohort of 3720 urban-dwelling African American and white adults recruited from 13 neighborhoods in Baltimore City, Maryland. Participants were between the ages of 30 and 64 years old at baseline (wave 1, 2004–2009) [100]. The study protocol was approved by the Institutional Review Board at the National Institute of Environmental Health Sciences. All participants provided written informed consent. For this study, participant data were taken from wave 1. Of the African American participants who met study recruitment criteria, we excluded individuals from the current analyses if they had a medical history of HIV/AIDS, were renal dialysis patients, did not fast prior to blood draws, or were missing data on any variables of interest. We used a complete case analysis for this cross-sectional study; missing data were not imputed. The final sample included 815 African American adults.

### Measures

#### Sociodemographic Characteristics and Covariates

Sex was defined as the sex assigned at birth (reference: women). SES was a dichotomous composite variable including poverty status, defined as an annual household income above or below 125% of the 2004 Federal poverty level relative to family size, and educational attainment vis-à-vis years in education. Participants considered above the poverty level and with ≥ 12 years of education (i.e., earned at least a high school diploma or GED) were classified as having higher SES. Those who were either below the poverty line or had < 12 years of education, or both, were classified as having lower SES (reference: higher SES; for additional review, see Waldstein et al. [101]). The use of antihypertensives, antidiabetic, or antilipidemic agents was self-reported and recoded into a single dichotomous variable reflecting medication use (reference: no treatment). Participants provided information on their faith tradition and/or denomination with fill-in responses, which were reviewed and reclassified into the following categories: (1) unaffiliated; (2) Christian or Catholic; (3) Islam, (4) Judaism; (5) others (e.g., Buddhism, etc.); and (6) illegible/indecipherable. These were reported for descriptive purposes only.

##### Outcome Variables

Systolic and diastolic BP were collected using a standard brachial artery auscultation method in the seated position; two measures across a 5-min time interval, one from each arm, were then averaged (mmHg). BMI was calculated by dividing weight in kilograms by height in meters squared (kg/m^2^), with measurements taken via calibrated equipment. Fasting blood tests were drawn to measure serum levels of glycated hemoglobin (HbA1c) and total cholesterol. Cholesterol was derived using a spectrophotometer (mg/dL), and HbA1c (%) was measured by way of liquid chromatography.

##### Predictor Variable

Racial discrimination was measured using a six-item instrument originally tested and validated in a large, epidemiological cohort study, which was also included in the Experiences of Discrimination Scale [102]. It included the following questions and domains: “Have you ever experienced racial discrimination (1) at school, (2) when getting a job, (3) at work, (4) when getting housing, (5) when getting medical care, and (6) from the police or in judicial courts?” (Cronbach’s *α* = 0.81). Almost half of the participants reported never experiencing racial discrimination (47.6%). A dichotomous variable was then created to reflect “no prior racial discrimination” (reference) versus “any experienced racial discrimination.”

##### Moderator Variable

Religious coping use comprised two items taken from the Religion subscale in the Brief COPE Inventory: “When confronted with a difficult or stressful event, I try to (1) find comfort in my religion or spiritual beliefs; and (2) pray or meditate” [103]. Responses ranged from 1 (“not at all”) to 4 (“a lot”). Higher scores indicated frequent religious coping use. Prior work has found this subscale to demonstrate high internal consistency and the overall inventory’s test–retest reliability to be stable [104, 105, 106]. In our study population, this scale had strong internal consistency among African American adults (Cronbach’s *α* = 0.75). Religious coping was mean-centered prior to regression analyses.

##### Sensitivity Variables

Depressive symptoms were characterized using the Center for Epidemiological Studies-Depression scale (CES-D) [107], which assessed depressive symptoms within the past week. Marital status was classified as either married/partnered versus single (reference). Instrumental and emotional social support coping use as well as substance use coping were also subscales taken from the Brief COPE Inventory [103]. Responses were summed per each dimension, as previously described for the moderator variable, and were standardized prior to analyses (Cronbach’s *α* = 0.70, 0.64, and 0.83, respectively). Cigarette, alcohol, and illicit drug use (marijuana, opiates, cocaine) were three separate dichotomous variables reflecting self-reported history of use (“ever used” versus “never used”: reference). Participants also self-reported previous diagnoses of the following CVDs—stroke or transient ischemic attack, coronary artery disease, coronary heart disease, claudication, heart attack/myocardial infarction, atrial fibrillation, or congestive heart failure. The medical history of prior CVDs was recoded to reflect “no diagnosis” (reference) or “any of these conditions.” Participants also indicated whether they had health insurance (uninsured: reference).

### Data Analytic Plan

Statistical analyses were conducted using R software version 4.4.0 [108]. Participant characteristics were described overall and stratified by sex. Student’s *t* tests and Chi-squared tests (*χ*^2^) were used to compare group means for continuous and categorical variables, respectively. Histograms and Q–Q plots were used to assess the normality of outcome variable distributions. Logarithmic data transformations were used to resolve skewness for HbA1c and BMI. All tests were two-tailed. A probability value of < 0.05 was considered statistically significant.

Hierarchical-entry, sex-stratified linear regression models were used to examine both the main effects and two-way interactive effects of racial discrimination and religious coping with respect to each CVD risk factor (systolic and diastolic BP, HbA1c, BMI, cholesterol) as outcome variables (i.e., parallel analyses were conducted in men and women). All base models controlled for sociodemographic variables (age, SES, medication use). Multicollinearity was assessed for each set of regression analyses. Two successive models were run for each CVD risk factor. In the first step, the main effects of racial discrimination and religious coping as well as sociodemographic covariates were included in the regression model (model 1). In the second step, the two-way interaction term (i.e., racial discrimination × religious coping) was added and assessed for its role in the respective CVD risk factor as the outcome (model 2). If the two-way interaction term was significant, interactive plots were produced and the main effects in model 1 were not interpreted. However, if the two-way interaction term did not reach statistical significance, then the main effects from model 1 were interpreted and retained as the final model.

After interactive plotting, the two-way interaction term was then decomposed using simple slope regressions to determine if the relationship between the frequency of religious coping use and the CVD risk factor varied by way of experienced discrimination (i.e., “any” versus “none”) and if the effect was statistically significant. Sensitivity analyses then assessed if the two-way interactive effect was independent of psychological, biobehavioral, social, and biomedical factors in individually clustered groupings. These included: (1) depressive symptoms; (2) cigarette, alcohol, illicit drug use, and substance use coping; (3) marital status, instrumental and emotional social support coping; and (4) medical history of prior CVDs, and health insurance status. We also conducted sensitivity testing with BMI in models that did not examine it as an outcome variable. We entered each set of clustered sensitivity variables into separate regression analyses to compensate for potentially reduced statistical power.

## Results

Sample descriptive characteristics of the final sample (*N* = 815 African American participants; 55.2% women, mean age = 48.61 years old, 57.7% low SES) can be found in Table 1. Overall, women were over-represented in this study sample (*p* < 0.001). More than half of all participants (52.3%) reported having previously experienced racial discrimination. More men than women endorsed these experiences. Men were also more likely to be married/partnered, smoke cigarettes, use illicit drugs, drink alcohol, and use substances as a means of coping compared to women. Conversely, women used religious coping more frequently than men as well as emotional social support coping. They were also more likely to have health insurance, currently use medication to manage CVD risk (antihypertensives, antidiabetics, antilipidemic agents), have hypertension, have higher BMI, and have lower DBP compared to men.

**Table 1:** Participant demographic characteristics and descriptive statistics for study variables: differences by sex

	Total sample (n = 815)	Women (*n* = 450)	Men (*n* = 365)	Sig
Sex (% men)	44.8	–	–	***
Age (years (± SD))	48.61 (9.16)	48.76 (9.18)	48.44 (9.15)	
SES (% high)	42.3	42.2	42.5	
Marital status (% single)	59.3	65.6	51.5	***
Emotional social support^a^ (± SD)	2.70 (1.72)	2.89 (1.71)	2.47 (1.70)	**
Instrumental social support^a^ (± SD)	2.95 (1.76)	2.93 (1.78)	2.96 (1.73)	
Cigarette use (% ever used)	67.9	61.6	75.6	***
Alcohol use (% ever used)	81.1	74.7	89.0	***
Illicit drug use (% ever used)	51.8	38.4	68.2	***
Substance use coping^a^ (± SD)	1.05 (1.85)	0.89 (1.71)	1.26 (2.00)	**
Previously diagnosed with CVD(s) (%)	167 (20.5)	22.4	18.1	
Medication use (% yes)	38.8	43.8	32.6	**
Health insurance (% yes)	63.9	67.6	59.5	*
Depressive symptoms^b^ (± SD)	14.24 (10.66)	14.61 (11.25)	13.79 (9.88)	
Systolic BP (mmHg (± SD))	121.23 (16.95)	121.36 (18.08)	121.08 (15.47)	
Diastolic BP (mmHg (± SD))	73.02 (10.97)	72.16 (10.71)	74.07 (11.21)	*
Body mass index (BMI; kg/m^2^ (± SD))	29.99 (7.77)	31.98 (8.44)	27.54 (6.03)	***
Glycated hemoglobin (% (± SD))	6.13 (1.36)	6.17 (1.38)	6.07 (1.34)	
Total cholesterol (mg/dL (± SD))	185.14 (42.33)	187.76 (38.14)	181.92 (46.85)	
Racial discrimination^c^ (% reported any)	52.4	43.6	63.3	***
Religious/Spiritual coping^a^ (% high)	44.3	51.8	35.1	***
Faith tradition (%)				***
Christian/Catholic	56.8	65.8	45.8	
Islam	2.8	1.1	4.9	
Judaism	0.1	0.0	0.3	
Other (Buddhism, etc.)	0.3	0.6	0.0	
Not affiliated with a religion	39.4	31.8	48.8	
Indecipherable^d^	0.5	0.7	0.3	

Healthy Aging in Neighborhoods of Diversity across the Life Span Study (HANDLS Study; *n* = 815; wave 1, 2004–2009). Values are presented as mean (± SD) unless otherwise indicated. Significant mean differences across sexes were examined with Student’s *t* tests and Chi-square tests of independence

**Abbreviations and notations:*** SD*, standard deviation; *SES*, socioeconomic status; *CVD(s)*, cardiovascular disease(s); *BP*, blood pressure

^*^p < 0.05; **p < 0.01; ***p < 0.001

^a^Brief-Cope Inventory (Carver, 1997)

^b^Center for Epidemiological Studies-Depression scale (107)

^c^Racial discrimination (102)

^d^Participants’ self-reported denominations were indecipherable but still included in analyses

Table 2 summarizes the results from the hierarchical-entry linear regressions among the male and female participants in the sample, with systolic and diastolic BP, HbA1c, BMI, and total cholesterol as separate outcomes, racial discrimination and religious coping as predictors, and age, SES, and medication use as covariates. The unstandardized regression coefficients are presented in Table 2 for all primary models that assessed the main effects and two-way interaction terms in sex-stratified analyses.

**Table 2:** Regression coefficients from multiple linear regression models estimating 2-way interactions for racial discrimination × religious coping with cardiovascular disease risk factors in sex-stratified analyses (HANDLS Study, *N* = 815)

*Variables*	Systolic blood pressure		Diastolic blood pressure		Glycated hemoglobin (HbA1c)		Body mass index		Cholesterol	
	Model 1	Model 2	Model 1	Model 2	Model 1	Model 2	Model 1	Model 2	Model 1	Model 2
African American men (*n* = 365)										
Age	0.34***	0.31**	0.01	− 0.01	0.00	0.00	0.00	0.00	0.01	− 0.05
Socioeconomic status^a^	0.47	0.40	1.20	1.16	0.02	0.02	0.08***	0.08***	− 1.80	− 1.98
Medication use^b^	4.73**	4.81**	2.97*	3.02*	0.16***	0.16***	0.13***	0.13***	− 4.47	− 4.27
Racial discrimination^c^	1.37	2.69	1.86	2.70*	0.00	0.01	0.03	0.04	6.00	9.36
Religious coping	0.79	− 0.89	1.67**	0.59	0.01	0.00	0.02	0.01	0.55	− 3.76
Racial discrimination × religious coping	–	4.39**	–	2.80*	–	0.04*	–	0.02	–	11.22*
African American women (*n* = 450)										
Age	0.70***	0.70***	0.09	0.08	0.00**	0.00**	0.00*	0.00*	0.39	0.40
Socioeconomic status^a^	− 3.10	− 3.06	− 2.16*	− 2.12*	− 0.03	− 0.03	0.03	0.03	5.85	5.79
Medication use^b^	4.81**	4.80**	2.15	2.14	0.10***	0.10***	0.19***	0.19***	7.21	7.23
Racial discrimination^c^	0.95	1.16	− 0.14	0.06	0.02	0.03	− 0.01	0.00	− 5.55	− 5.82
Religious coping	0.49	1.16	0.14	0.80	0.00	0.02	0.00	0.02	3.12	2.27
Racial discrimination × religious coping	–	− 1.18	–	− 1.16	–	− 0.04*	–	− 0.05*	–	1.48

The values presented are regression coefficients. Model 1 assessed the main effects and covariates. Model 2 assessed the 2-way interactive effect. A complete list of inferential statistics, including *p* values and *η*^2^, are listed in the Supplemental Files

^*^p < 0.05; ** *p* < 0.01; *** *p* < 0.001

^a^Low, reference group

^b^Not currently using any antihypertensive, antidiabetic, or antilipidemic agent or medication

^c^None, reference group

### Sex-Stratified Analyses: Results for African American Men

**Fig. 1:**
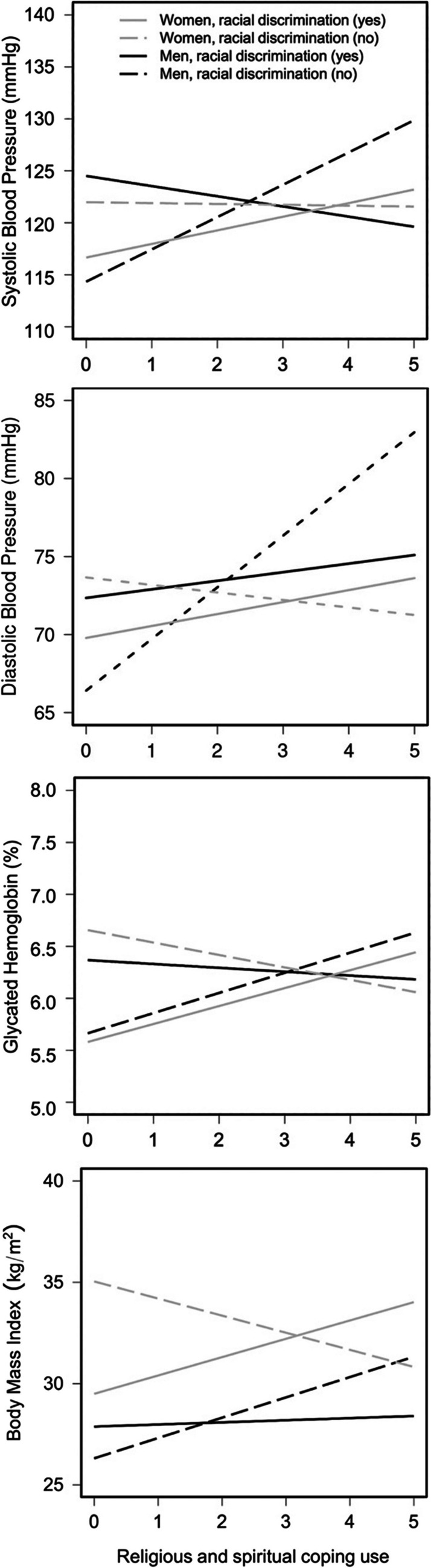
Interactive plots demonstrating associations of racial discrimination × religious coping with cardiovascular disease risk factors among African American men and women

In analyses examining systolic BP as the outcome, in the first step, Model 1 showed neither racial discrimination nor religious coping use were significant main effects, *F*(5, 359) = 6.64, *R*^2^ = 0.072, *p* < 0.001. However, the addition of the interaction term in the second step explained an additional 1.79% of the variance, *F*(6, 358) = 6.98, *R*^2^ = 0.090, *p* < 0.001, and was statistically significant (*b* = 4.39, SE = 1.55, *p* = 0.005, *η*^2^ = 0.02; see Model 2 in Table 2; the Supplemental File contains the full results of all regression models). As shown in Fig. 1, simple regression slopes showed that religious coping use was positively associated with systolic BP among men who reported no prior racial discrimination (*b* = 3.49, *p* < 0.01) but was inversely associated with systolic BP among men who experienced racial discrimination (*b* =  − 0.89, *p* = 0.35) (see Table 3 for full results).

**Table 3:** Simple regression slopes estimating the effects of religious coping use on predicting cardiovascular disease risk factors per experienced racial discrimination: parameter estimates for African American men (*n* = 365)

*Racial discrimination*	*Estimate* (*SE*)	95% confidence intervals		*t*	*p*
		Lower	Upper		
*Systolic blood pressure (model 2)*					
No prior experiences	3.49 (1.21)	1.11	5.88	2.88	< 0.01
Experienced racial discrimination	− 0.89 (0.96)	− 2.78	0.99	− 0.93	0.35
*Diastolic blood pressure (model 2)*					
No prior experiences	3.40 (0.90)	1.62	5.17	3.77	< 0.01
Experienced racial discrimination	0.59 (0.71)	− 0.81	2.00	0.83	0.40
*Glycated hemoglobin (model 2)*					
No prior experiences	0.03 (0.01)	0.01	0.06	2.46	0.01
Experienced racial discrimination	− 0.00 (0.01)	− 0.03	0.02	− 0.45	0.66
*Total cholesterol (model 2)*					
No prior experiences	7.46 (3.85)	− 0.11	15.02	1.94	0.05
Experienced racial discrimination	− 3.76 (3.04)	− 9.74	2.22	− 1.24	0.22

*SE*, standard error

In analyses examining diastolic BP as the outcome, in the first step, model 1 found a main effect of religious coping, *F*(5, 359) = 3.38, *R*^2^ = 0.032, *p* = 0.005. Greater religious coping was associated with higher levels of diastolic BP (*b* = 1.68, SE = 0.56, *p* = 0.003, *η*^2^ = 0.02; see model 1 in Table 2). In the second step, the addition of the interaction term explained an additional 1.32% of the variance, *F*(6, 358) = 3.85, *R*^2^ = 0.045, *p* < 0.001, and was also statistically significant (*b* = 2.80, SE = 1.15, *p* = 0.02, *η*^2^ = 0.02). Consequently, the significant main effect of religious coping in model 1 was no longer interpreted. As shown in Fig. 1, simple regression slopes showed that religious coping use was positively associated with diastolic BP for all men. However, the magnitude of these relations was smaller among those who reported experiencing racial discrimination (*b* = 0.59, *p* = 0.40) compared to men who reported no prior racial discrimination (*b* = 3.40, *p* < 0.01) (see Table 3 for full results).

In analyses examining HbA1c as the outcome, in the first step, similar to the findings for systolic BP, model 1 showed neither racial discrimination nor religious coping use were significant main effects, *F*(5, 359) = 15.10, *R*^2^ = 0.162, *p* < 0.001. However, the addition of the interaction term explained an additional 0.90% of the variance, *F*(6, 358) = 13.53, *R*^2^ = 0.171, *p* < 0.001, and was statistically significant (*b* = 0.04, SE = 0.02, *p* = 0.03, *η*^2^ = 0.01; see model 2 in Table 2). As shown in Fig. 1 and in Table 3, simple regression slopes showed that religious coping use was positively associated with HbA1c among men who reported no prior racial discrimination (*b* = 0.03, *p* = 0.01), but was unrelated to HbA1c among men who reported previously experiencing racial discrimination (*b* = 0.00, *p* = 0.66).

In analyses examining BMI as the outcome, in the first step, similar to the findings for systolic BP and HbA1c, model 1 showed neither racial discrimination nor religious coping use were significant main effects, *F*(5, 359) = 9.14, *R*^2^ = 0.101, *p* < 0.001, though religious coping was marginally associated with higher BMI (*b* = 0.02, SE = 0.01, *p* = 0.07, *η*^2^ = 0.009). However, in the second step, the addition of the interaction term did not explain any additional variance in the overall model, and it was not significant (*b* = 0.02, SE = 0.02, *p* = 0.32, *η*^2^ = 0.003; see model 2 in Table 2). No further analyses with BMI as the outcome variable were conducted.

Lastly, in analyses examining total cholesterol as the outcome, in the first step, similar to prior findings, neither racial discrimination nor religious coping use were significant main effects, but the overall model was also nonsignificant, *F*(5, 359) = 0.45, *R*^2^ =  − 0.008, *p* = 0.81. In the second step, although the addition of the interaction term explained an additional 1.18% of variance and was statistically significant (*b* = 11.22, SE = 4.90, *p* = 0.02, *η*^2^ = 0.01), the overall model fit remained nonsignificant, *F*(6, 358) = 1.26, *R*^2^ = 0.004, *p* = 0.278 (see model 2 in Table 2 and the Supplemental File for full results of the regression models). The interaction term was therefore rendered nonsignificant. Across all regression models for the five separate outcome variables, there were no issues of multicollinearity (VIF < 1.13).

### Sensitivity Testing: Results for African American Men

Sensitivity analyses were conducted for the significant interactive relationships only (systolic and diastolic BP, HbA1c). All findings remained significant after additional adjustments were made for the following sensitivity variables in clustered groupings: (1) depressive symptoms; (2) cigarette, alcohol, and illicit drug use, substance use coping; (3) marital status, instrumental and emotional social support coping; and (4) medical history of prior CVDs, health insurance status. When BMI was examined as a sensitivity variable, the two-way interactive effect remained significant for both systolic and diastolic BP but lost significance for HbA1c (*p* = 0.06). (These results can be found in the Supplemental File.)

### Sex-Stratified Analyses: Results for African American Women

In analyses examining systolic BP as the outcome, in the first step, model 1 showed neither racial discrimination nor religious coping use were significant main effects, *F*(5, 445) = 6.64, *R*^2^ = 0.177, *p* < 0.001. When the interaction term was added in the second step, no additional variance was explained, *F*(6, 444) = 17.01, *R*^2^ = 0.176, *p* < 0.001, and the interaction was not significant (*b* =  − 1.18, SE = 1.69, *p* = 0.48, *η*^2^ = 0.001; see model 2 in Table 2 and the Supplemental File for full results of the regression models).

In analyses examining diastolic BP as the outcome, in the first step, similar to the findings from systolic BP, model 1 showed neither racial discrimination nor religious coping use were significant main effects, *F*(5, 445) = 2.69, *R*^2^ = 0.018, *p* = 0.02. When the interaction term was added in the second step, an additional 0.03% of variance was explained, *F*(6, 444) = 2.43, *R*^2^ = 0.019, *p* = 0.03, but the interaction was not significant (*b* =  − 1.15, SE = 1.10, *p* = 0.29, *η*^2^ = 0.003; see model 2 in Table 2).

In analyses examining log-HbA1c as the outcome, in the first step, similar to previous findings, model 1 showed neither racial discrimination nor religious coping use were significant main effects, *F*(5, 445) = 13.08, *R*^2^ = 0.118, *p* < 0.001. In the second step, the addition of the interaction term explained an additional 0.80% of the variance, *F*(6, 444) = 13.53, *R*^2^ = 0.171, *p* < 0.001, and was statistically significant (*b* =  − 0.04, SE = 0.02, *p* = 0.02, *η*^2^ = 0.01; see model 2 in Table 2). As shown in Fig. 1, simple regression slopes showed that religious coping use was negatively associated with log-HbA1c among women who reported no prior racial discrimination (*b* =  − 0.01, *p* = 0.23) but was positively associated with log-HbA1c among women who reported previously experiencing racial discrimination (*b* = 0.02, *p* = 0.06) (see Table 4 for full results).

**Table 4:** Simple regression slopes estimating the effects of religious coping use on predicting cardiovascular disease risk factors per experienced racial discrimination: parameter estimates for African American women (*n* = 450)

*Racial discrimination*	*Estimate* (*SE*)	95% confidence intervals		*t*	*p*
		Lower	Upper		
*Glycated hemoglobin (model 2)*					
No prior experiences	− 0.01 (0.01)	− 0.04	0.01	− 1.21	0.23
Experienced racial discrimination	0.02 (0.01)	− 0.00	0.05	1.92	0.06
*Body mass index (model 2)*					
No prior experiences	− 0.03 (0.02)	− 0.06	0.01	− 1.57	0.12
Experienced racial discrimination	0.02 (0.02)	− 0.01	0.06	1.24	0.22

*SE*, standard error

In analyses examining log-BMI as the outcome, in the first step, similar to previous findings, model 1 showed neither racial discrimination nor religious coping use were significant main effects, *F*(5, 445) = 10.66, *R*^2^ = 0.097, *p* < 0.001. In the second step, the addition of the interaction term explained an additional 0.59% of the variance, *F*(6, 444) = 9.59, *R*^2^ = 0.103, *p* < 0.001, and was statistically significant (*b* =  *− *0.05, SE = 0.02, *p* = 0.049, *η*^2^ = 0.009; see model 2 in Table 2). As shown in Fig. 1 and in Table 4, simple regression slopes showed that religious coping use was negatively associated with log-BMI among women who reported no prior racial discrimination (*b* =  − 0.03, *p* = 0.12) but was positively associated with log-BMI among women who reported previously experiencing racial discrimination (*b* = 0.02, *p* = 0.22).

Finally, in analyses examining total cholesterol as the outcome, in the first step, similar to previous results, model 1 showed neither racial discrimination nor religious coping use was the significant main effect, *F*(5, 445) = 4.62, *R*^2^ = 0.039, *p* < 0.001. When the interaction term was added in the second step, an additional 0.18% of the variance was explained: *F*(6, 444) = 3.87, *R*^2^ = 0.037, *p* < 0.001, but the interaction term was not significant (*b* = 1.48, SE = 3.85, *p* = 0.70, *η*^2^ = 0.003; see model 2 in Table 2). Across all regression models for the five separate outcome variables, there were no issues of multicollinearity (VIF < 1.23).

### Sensitivity Testing: Results for African American Women

Sensitivity analyses were also conducted only for the significant associations found in African American women (HbA1c, BMI). Similar to the sensitivity testing done in men, the findings for log-HbA1c as an outcome remained significant after adjustments were made for (1) depressive symptoms; (2) cigarette, alcohol, and illicit drug use and substance use coping; (3) marital status and instrumental and emotional social support coping; and (4) medical history of prior CVDs and health insurance status. However, when BMI was examined as a sensitivity variable, the interactive effect lost significance (*p* = 0.05). In models examining log-BMI as the outcome variable, findings withstood adjustment for depressive symptoms and biomedical (medical history of prior CVDs, health insurance status) factors. However, the two-way interaction term lost significance when models adjusted for biobehavioral factors (cigarette, alcohol, and illicit drug use and substance use coping; *p* = 0.10) and social support indicators (marital status and instrumental and emotional social support coping* p* = 0.05). These results can be found in the Supplementary File.

### Exploratory Analyses: Combined-Sample Moderation Results

In the overall sample of adults, we reran analyses and tested up to the three-way interactive effect of racial discrimination × religious coping × sex with the five biological measures of CVD risk. There were four significant three-way interactions among (1) systolic BP (*b* = 5.47, *p* = 0.02), (2) diastolic BP (*b* = 4.01, *p* = 0.01), (3) log-HbA1c (*b* = 0.08, *p* = 0.002), and (4) log-BMI (*b* = 0.07, *p* = 0.03). These results can be found in the Supplemental File.

## Discussion

An emerging body of work has proposed that religious coping acts as a “stress-buffering resource,” wherein the expected health detriments associated with a given stressor (experienced discrimination) are lessened, in part, because of coping behaviors and strategies tied to religion. Less is known about how these moderating effects influence modifiable CVD risk factors for African American adults and if these associations vary by sex. Our cross-sectional study found that among men who experienced racial discrimination, greater religious coping use seemingly diminished the adverse effects associated with racial discrimination on some CVD risk factors (BP, HbA1c). However, men who reported never having experienced discrimination but used religious coping frequently showed elevated levels for most CVD risk factors. These relationships were independent of other sociodemographic characteristics and psychological biobehavioral, social support, and biomedical factors, except BMI. Contrastingly, for women, no buffering effects were found. Rather, higher levels of HbA1c and BMI were observed among those who experienced racial discrimination and endorsed frequent religious coping. These associations, however, lost significance when BMI as well as biobehavioral and social support factors were considered. Our primary findings suggest that frequent engagement in religious coping behaviors may reduce the potentially pernicious effects of race-related stress on poorer cardiovascular health for African American men.

To date, only one prior study has examined the interactive associations of unfair treatment and religious coping use with incident hypertension but found no significant moderating effects [66]. Methodological inconsistencies with this prior study may partially explain their null findings. Namely, their participant data were drawn from a study of white and black (African American and Caribbean) Seventh Day Adventists, and the outcome assessed was incident hypertension rather than BP levels. Here, we only examined these relationships within African American adults, given that their experiences with racial discrimination are much more salient and qualitatively divergent from those of white Americans [9, 109]. Also, the stark racial variations across religiosity and the historical relevance of black-affirming religious spaces in a racially marginalizing society suggested that examining these relationships would be most applicable to the African American community.

Our study found these associations to be sex-specific, in that among those who previously experienced racial discrimination, greater religious coping use demonstrably proved to mitigate poorer cardiovascular health for African American men but not women. Prior studies have pointed to possible sex differences across the interactive relations of psychosocial stress/risk and resiliency factors with cardiovascular and overall health outcomes for African American adults [110, 111, 112]. Yet still, it remains unclear if these associations are more striking for either women or men. Engaging in harmful behavioral health-related coping activities like substance use and other unhelpful active coping styles like John Henryism (beliefs and strategies that one can overcome racism by working harder and longer) have been linked with poorer health outcomes and increased CVD risk for African American men, whereas positive, protective factors like optimism and resilience have proved exclusively advantageous for men, too [113, 114, 115, 116, 117]. Active involvement in faith-based communities and religious teachings in black-affirming churches can heavily influence African American men’s racial socialization, developmental processes, and empowerment. When they have encountered racial discrimination, these positive perceptions of leadership, fathering roles, and masculinity are encouragingly helpful [118, 119]. African American men are often afflicted with more severe and fatal clinical CVDs and comorbid conditions and are less likely to maintain routine visits with their primary healthcare providers or manage CVD risk well [120, 121]. Future research should continue to investigate how the interplay of psychosocial risk-and-resiliency factors for African American men affects their long-term cardiovascular health and their risk for severer progression of CVDs.

When faced with race-related stress, religious African American adults turn to religious coping strategies to make sense of what has happened [45, 122, 123]. Repeated exposure to racial discrimination can result in long-term wear and tear on the body and adverse cardiovascular health outcomes [14, 124, 125]. Religious practices, such as prayer or seeking church-based social support, provide ways to cope with and address stressful situations like discrimination [45]. African American church communities also try to encourage mindfulness-based practices and health-promoting behaviors as a way to combat racial health disparities [126, 127, 128]. Our findings contribute to the nascent literature that the physiological burden of racial discrimination might be lessened for African American men who turn to religion as a coping resource. The explicit mechanisms underlying the relationships between racial discrimination, religion, and CVD risk remain understudied.

Notably, though, two additional peculiarities arose from our findings. In the absence of discrimination, men who endorsed greater religious coping use had higher levels across most CVD risk factors examined (Fig. 1). A couple of explanations could potentially clarify these findings. It is possible that other chronic psychosocial or environmental stressors that were not accounted for in this study are affecting their overall cardiovascular health (e.g., workplace stress; [129]). In like manner, there may be some bidirectionality, wherein men suffering from comorbid conditions (e.g., hypertension, diabetes) turn to religion to cope with their health concerns [87]. Also, religious coping use is highly correlated with other indicators of religious involvement; thus, we presume that these participants might also be religious individuals [82, 130, 131]. Prior reports have noted higher rates of CVD-associated comorbidities (hypertension, obesity, diabetes), and clinical events were observed among more religious African American men and others (frequent church attendees), despite religion being commonly thought of as a protective factor related to optimal health outcomes [83, 87]. African American men who are overly committed and involved in church leadership and community-related activities may have less time and energy for health-promoting lifestyle behaviors or might be less motivated to address health conditions [132]. Additionally, sometimes church-sponsored events feature high-caloric foods, and black pastors may avoid discussing medical issues from the pulpit for fear of stigmatizing people or due to a lack of knowledge, confidence, or awareness of the community’s health needs [133].

In addition, some forms of religious coping (e.g., deferred religious coping) may be ineffective in addressing health issues if they are not partnered with more health-conscious behaviors, such as following physician advice or medication adherence [134]. Existing research notes that African American men may defer both health issues and experiences with racism to a higher power [89]. This could contribute to their under-reporting past experiences of racial discrimination. However, when paired with a high reliance on religious coping, this could lead to elevated CVD risk. Studies have highlighted that religion might have a “dark side,” in that it is not always advantageous for emotional regulation and physical wellbeing. Sometimes, religious people may avoid directly dealing with a stressful situation or a major health concern because they believe their divine power will handle it for them, or their religious coping manifests as excessive worrying, self-imposed blame (i.e., what is happening to them is their fault), or religious fatalism (i.e., what is happening to them is divinely ordained) [86, 135, 136]. The nuances of religious coping and religious involvement as a form of coping within the African American community merit further attention in future research.

At the same time, some studies have also surprisingly found inverse relations with respect to discrimination and health outcomes, wherein African American men who reported no discrimination fared worse with respect to their health [137, 138]. Researchers surmise alternative pathways of discrimination appraisal, suggesting that some African American adults may be under-reporting prior experiences of unfair treatment due to memory suppression [139]. For some African American men, admitting they were treated unfairly because of their race might be hard to express due to other personal characteristics (i.e., pride), cultural sways, or simply because they expect it to happen [90]. To this end, it is also possible that since most religions encourage forgiveness, religious African American men may also suppress these memories and emotions (i.e., forgive and *forget*) even if the harm committed against them still stings. The health detriments associated with these forms of stress exposure can still manifest regardless. Our findings further reinforce the need for attention to examine how the interplay of these psychosocial determinants affects African American men’s cardiovascular health overall. There are dire implications when determinants are singularly viewed as protective or risk factors.

Similarly, our second peculiar finding was that religious coping did not buffer the associations between racial discrimination and CVD risk factors among African American women. African American women’s use of religious coping to deal with a broad range of personal, health-related, and social stressors has been linked with better emotional and physical wellbeing [29, 51, 111, 123, 140]. Scholars have discussed how religion impacts black women’s self-perceptions, motivations, and coping behaviors [123, 141, 142]. However, these “anchors” can also lead to unique social expectations or self-sacrificing behaviors that blur the lines of coping strategies. The *Strong Black Woman Schema*, for example, is grounded in endurance through intersectional oppression, often referencing religious ideologies [91]. However, it is still complex. While the Strong Black Woman Schema intimates inner strength and divine hope as resilience, it also pushes for self-determination and perseverance through overcoming adversity, which can be emotionally challenging and physiologically harmful for some women, too [143, 144]. We were unable to distinguish between resilience and positive or negative religious coping sentiments in this study, but these remain thought-provoking questions that require further attention [145].

Furthermore, some interpretations of sacred texts can shape narratives of what it means to suffer and how to endure suffering, especially in the face of social adversity [146]. Despite its renowned legacy of fighting against racial inequality, the institutional black church has also been silent on, or perpetuated, other forms of injustice like sexism [141, 147]. For instance, a national survey report conducted by the Pew Research Center showed that nearly half of black Protestants who attend predominantly black churches heard sermons about racism (47%), whereas less than one-third heard sermons about sexism (31%) [36]. African American women contribute greatly to their religious communities as well as organizational events and related activities, often voluntarily. But by and large, they hold fewer official positions of leadership and power, even though they comprise the majority of most religious congregations [148]. Whereas most literature predominantly included Christian-majority samples, studies exclusively featuring African American Muslim women also demonstrate the centrality of faith and community when confronted with discrimination [41, 149, 150]. The lack of buffering effects in this study was surprising but suggests that either religious coping use is not always beneficial for African American women, despite their consistently higher religious profiles, or there may be concerns about measurement issues. Religious coping measures might not fully capture the intricate interactions and diverse experiences of African American women at church or in their communities. Additionally, by focusing on racial discrimination, our study’s results may be obscured for African American women who experience *gendered* racism, sexism, and other salient forms of discrimination. Future studies should incorporate discrimination measurements that better attend to intersectional identities and continue to examine variations across these linkages before assessing these associations as equivalent for women and men. Our findings corroborate that the health effects of risk and resilience factors need to be studied in African American women and men separately.

Correspondingly, this study had other limitations that required acknowledgment. First, the analyses were cross-sectional, so we were precluded from determining temporality. Mediation was also not tested, given the nature of the study’s parameters, so it is also unclear if any intermediary variables partially explain these relationships (e.g., BMI, health behaviors, social support indicators). Replica studies are needed to confirm if religious coping use bestows buffering effects over time and why these associations might be sex-specific. Second, discrimination is both multidimensional (e.g., intersectional, sex-based, and race-related) and multilevel (e.g., structural, vicarious, or “second-hand”). It is possible that different dimensions of discrimination may presage deviating biological underpinnings. Future work should assess other levels and aspects of discrimination that contribute to poorer health among African American adults [4, 16, 151, 152]. Coupled with this, our measurement of religious coping use only comprised two items, and we were unable to extrapolate which specific coping styles temper or exacerbate the damaging effects of discrimination on CVD risk for African American adults (e.g., positive versus negative coping, self-directing coping styles, meditation rather than prayer). Future work should explore other dimensions of religious coping as well as religious participation and spirituality to determine which behaviors blunt discrimination’s effects on health outcomes and which are most helpful for African American men and women when confronted with race-related stress and mistreatment.

## Conclusion

The current study contributes to the existing literature by examining the potential buffering effects of religious coping use on the relations between racial discrimination and CVD risk among a sample of urban-dwelling, midlife African American women and men. Here, we found that for African American men who experienced prior racial discrimination, higher religious coping use was related to diminished CVD risk, but these anticipated effects were not seen among men in the absence of discrimination nor among women who experienced discrimination. Taken together, it remains exceedingly important to consider the continued relevance of religious coping as a mechanism for ameliorating health disadvantages, especially for African American men, for whom the risk of CVD comorbidities and severer and fatal CVDs remains high. Additional work is warranted to further elucidate the mechanisms underlying how psychosocial determinants like racial discrimination and religion “get under the skin.” Moreover, our study confirms that individual- and community-level interventions must attend to the social conditions and culturally lived experiences of African American women and men uniquely. There is an increased reliance on and interest in partnering with predominantly black faith-based communities to achieve large-scale health promotion efforts. These collaborations are also viable opportunities for researchers to identify helpful coping behaviors to prevent exacerbated poorer health due to racism and racial discrimination. Clinicians and medical practitioners can also use psychosocial history questionnaires to assess individuals’ interpersonal problems, direct them to community-based initiatives and resources, or inspire patients to use their intraindividual strategies to help them cope.

## Replication

The HANDLS sample is relatively small and drawn from a vulnerable population residing in specific census tracts in Baltimore City, Maryland, USA. Therefore, maintaining confidentiality—especially in the context of a longitudinal study—is paramount. Participants’ identities are at risk under these conditions. Therefore, interested investigators should consult the HANDLS Website at https://handls.nih.gov-specifically, the instructions for collaborators at https://handls.nih.gov/06Coll-dataDoc.htm. Questions should be directed to Alan Zonderman at zondermana@mail.nih.gov.

## Supplementary Information

Below is the link to the electronic supplementary material.

Supplementary file1 (DOCX 116 KB)
