# Supplementary material for: Racial Discrimination, Religious Coping, and Cardiovascular Disease Risk Among African American Women and Men
Source: J Racial Ethn Health Disparities. Author manuscript; Available in PMC 2025 Sep 19. (PMC11954130; doi:10.1007/s40615-024-02113-x)
Supplement: Supplementary file 1 [file NIHMS2055299-supplement-Supplementary_files.docx]

**SUPPLEMENTARY TABLES & RELATED MATERIAL**

| **Table 1.** Interactive Associations between Racial Discrimination and Religious Coping Predicting Systolic Blood Pressure in African American Men, HANDLS Study (N = 365) |  |  |  |  |  |  |  |  |  |  |
| --- | --- | --- | --- | --- | --- | --- | --- | --- | --- | --- |
|  | Model 1 |  |  |  |  | Model 2 |  |  |  |  |
| *Variables* | *b* | SE | *t* | *p* | η^2^ | *b* | SE | *t* | *p* | η^2^ |
| Age | 0.34*** | 0.09 | 3.70 | .000 | .060 | 0.31** | 0.09 | 3.46 | .001 | .070 |
| Socioeconomic status | 0.47 | 1.58 | 0.30 | .766 | .000 | 0.40 | 1.57 | 0.26 | .798 | .000 |
| Medication use | 4.73** | 1.76 | 2.68 | .008 | .020 | 4.81** | 1.74 | 2.76 | .006 | .020 |
| Racial discrimination | 1.37 | 1.65 | 0.83 | .405 | .001 | 2.69 | 1.69 | 1.59 | .114 | .001 |
| Religious coping | 0.79 | 0.76 | 1.04 | .299 | .003 | -0.89 | 0.96 | -0.93 | .353 | .003 |
| Racial discrimination × Religious coping |  |  |  |  |  | 4.39** | 1.55 | 2.84 | .005 | .020 |
| **Note**. * *p* < .05, ** *p* < .01, *** *p* < .001. **Abbreviations**. HANDLS Study = Healthy Aging in Neighborhoods of Diversity across the Life Span Study. SE = Standard error. Socioeconomic status = low, reference group. Medication use = Not currently using any antihypertensive, antidiabetic, or antilipidemic agent or medication. Racial discrimination = none, reference group. |  |  |  |  |  |  |  |  |  |  |

| **Table 2.** Interactive Associations between Racial Discrimination and Religious Coping Predicting Diastolic Blood Pressure in African American Men, HANDLS Study (N = 365) |  |  |  |  |  |  |  |  |  |  |
| --- | --- | --- | --- | --- | --- | --- | --- | --- | --- | --- |
|  | Model 1 |  |  |  |  | Model 2 |  |  |  |  |
| *Variables* | *b* | SE | *t* | *p* | η^2^ | *b* | SE | *t* | *p* | η^2^ |
| Age | 0.01 | 0.07 | 0.09 | .928 | .004 | -0.01 | 0.07 | -0.13 | .897 | .004 |
| Socioeconomic status | 1.20 | 1.17 | 1.02 | .306 | .002 | 1.16 | 1.16 | 0.99 | .321 | .002 |
| Medication use | 2.97* | 1.30 | 2.28 | .023 | .010 | 3.02* | 1.29 | 2.33 | .020 | .010 |
| Racial discrimination | 1.86 | 1.22 | 1.53 | .127 | .003 | 2.70* | 1.26 | 2.15 | .032 | .003 |
| Religious coping | 1.67** | 0.56 | 2.96 | .003 | .020 | 0.59 | 0.71 | 0.83 | .404 | .020 |
| Racial discrimination × Religious coping |  |  |  |  |  | 2.80* | 1.15 | 2.44 | .015 | .020 |
| **Note**. * *p* < .05, ** *p* < .01, *** *p* < .001. **Abbreviations**. HANDLS Study = Healthy Aging in Neighborhoods of Diversity across the Life Span Study. SE = Standard error. Socioeconomic status = low, reference group. Medication use = Not currently using any antihypertensive, antidiabetic, or antilipidemic agent or medication. Racial discrimination = none, reference group. |  |  |  |  |  |  |  |  |  |  |

| **Table 3.** Interactive Associations between Racial Discrimination and Religious Coping Predicting Glycated Hemoglobin (HbA1c) in African American Men, HANDLS Study (N = 365) |  |  |  |  |  |  |  |  |  |  |
| --- | --- | --- | --- | --- | --- | --- | --- | --- | --- | --- |
|  | Model 1 |  |  |  |  | Model 2 |  |  |  |  |
| *Variables* | *b* | SE | *t* | *p* | η^2^ | *b* | SE | *t* | *p* | η^2^ |
| Age | 0.00 | 0.00 | -0.35 | .725 | .020 | 0.00 | 0.00 | -0.55 | .581 | .020 |
| Socioeconomic status | 0.02 | 0.02 | 1.20 | .231 | .002 | 0.02 | 0.02 | 1.17 | .242 | .002 |
| Medication use | 0.16*** | 0.02 | 8.14 | .000 | .160 | 0.16*** | 0.02 | 8.22 | .000 | .160 |
| Racial discrimination | 0.00 | 0.02 | -0.17 | .865 | .000 | 0.01 | 0.02 | 0.44 | .659 | .000 |
| Religious coping | 0.01 | 0.01 | 1.17 | .244 | .004 | 0.00 | 0.01 | -0.45 | .655 | .004 |
| Racial discrimination × Religious coping |  |  |  |  |  | 0.04* | 0.02 | 2.21 | .027 | .010 |
| **Note**. * *p* < .05, ** *p* < .01, *** *p* < .001. **Abbreviations**. HANDLS Study = Healthy Aging in Neighborhoods of Diversity across the Life Span Study. SE = Standard error. Socioeconomic status = low, reference group. Medication use = Not currently using any antihypertensive, antidiabetic, or antilipidemic agent or medication. Racial discrimination = none, reference group. |  |  |  |  |  |  |  |  |  |  |

| **Table 4.** Interactive Associations between Racial Discrimination and Religious Coping Predicting Body Mass Index in African American Men, HANDLS Study (N = 365) |  |  |  |  |  |  |  |  |  |  |
| --- | --- | --- | --- | --- | --- | --- | --- | --- | --- | --- |
|  | Model 1 |  |  |  |  | Model 2 |  |  |  |  |
| *Variables* | *b* | SE | *t* | *p* | η^2^ | *b* | SE | *t* | *p* | η^2^ |
| Age | 0.00 | 0.00 | -1.82 | .070 | .000 | 0.00 | 0.00 | -1.90 | .058 | .000 |
| Socioeconomic status | 0.08*** | 0.02 | 3.73 | .000 | .030 | 0.08*** | 0.02 | 3.72 | .000 | .030 |
| Medication use | 0.13*** | 0.02 | 5.40 | .000 | .070 | 0.13*** | 0.02 | 5.42 | .000 | .070 |
| Racial discrimination | 0.03 | 0.02 | 1.56 | .121 | .004 | 0.04 | 0.02 | 1.77 | .078 | .004 |
| Religious coping | 0.02 | 0.01 | 1.80 | .073 | .009 | 0.01 | 0.01 | 0.79 | .428 | .009 |
| Racial discrimination × Religious coping |  |  |  |  |  | 0.02 | 0.02 | 1.00 | .318 | .003 |
| **Note**. * *p* < .05, ** *p* < .01, *** *p* < .001. **Abbreviations**. HANDLS Study = Healthy Aging in Neighborhoods of Diversity across the Life Span Study. SE = Standard error. Socioeconomic status = low, reference group. Medication use = Not currently using any antihypertensive, antidiabetic, or antilipidemic agent or medication. Racial discrimination = none, reference group. |  |  |  |  |  |  |  |  |  |  |

| **Table 5.** Interactive Associations between Racial Discrimination and Religious Coping Predicting Cholesterol in African American Men, HANDLS Study (N = 365) |  |  |  |  |  |  |  |  |  |  |
| --- | --- | --- | --- | --- | --- | --- | --- | --- | --- | --- |
|  | Model 1 |  |  |  |  | Model 2 |  |  |  |  |
| *Variables* | *b* | SE | *t* | *p* | η^2^ | *b* | SE | *t* | *p* | η^2^ |
| Age | 0.01 | 0.29 | 0.04 | .971 | .000 | -0.05 | 0.29 | -0.17 | .865 | .000 |
| Socioeconomic status | -1.80 | 4.99 | -0.36 | .718 | .000 | -1.98 | 4.96 | -0.40 | .690 | .000 |
| Medication use | -4.47 | 5.55 | -0.81 | .421 | .002 | -4.27 | 5.52 | -0.77 | .440 | .002 |
| Racial discrimination | 6.00 | 5.19 | 1.16 | .249 | .004 | 9.36 | 5.37 | 1.75 | .082 | .004 |
| Religious coping | 0.55 | 2.40 | 0.23 | .820 | .000 | -3.76 | 3.04 | -1.24 | .217 | .000 |
| Racial discrimination × Religious coping |  |  |  |  |  | 11.22* | 4.90 | 2.29 | .023 | .010 |
| **Note**. * *p* < .05, ** *p* < .01, *** *p* < .001. **Abbreviations**. HANDLS Study = Healthy Aging in Neighborhoods of Diversity across the Life Span Study. SE = Standard error. Socioeconomic status = low, reference group. Medication use = Not currently using any antihypertensive, antidiabetic, or antilipidemic agent or medication. Racial discrimination = none, reference group. |  |  |  |  |  |  |  |  |  |  |

| **Table 6.** Interactive Associations between Racial Discrimination and Religious Coping Predicting Systolic Blood Pressure in African American Women, HANDLS Study (N = 410) |  |  |  |  |  |  |  |  |  |  |
| --- | --- | --- | --- | --- | --- | --- | --- | --- | --- | --- |
|  | Model 1 |  |  |  |  | Model 2 |  |  |  |  |
| *Variables* | *b* | SE | *t* | *p* | η^2^ | *b* | SE | *t* | *p* | η^2^ |
| Age | 0.70*** | 0.09 | 7.47 | .000 | .170 | 0.70*** | 0.09 | 7.44 | .000 | .170 |
| Socioeconomic status | -3.10 | 1.58 | -1.96 | .051 | .007 | -3.06 | 1.59 | -1.93 | .055 | .007 |
| Medication use | 4.81** | 1.73 | 2.78 | .006 | .020 | 4.80** | 1.73 | 2.77 | .006 | .020 |
| Racial discrimination | 0.95 | 1.57 | 0.61 | .543 | .001 | 1.16 | 1.60 | 0.73 | .466 | .001 |
| Religious coping | 0.49 | 0.85 | 0.57 | .567 | .001 | 1.16 | 1.28 | 0.90 | .367 | .001 |
| Racial discrimination × Religious coping |  |  |  |  |  | -1.18 | 1.69 | -0.70 | .485 | .001 |
| **Note**. * *p* < .05, ** *p* < .01, *** *p* < .001. **Abbreviations**. HANDLS Study = Healthy Aging in Neighborhoods of Diversity across the Life Span Study. SE = Standard error. Socioeconomic status = low, reference group. Medication use = Not currently using any antihypertensive, antidiabetic, or antilipidemic agent or medication. Racial discrimination = none, reference group. |  |  |  |  |  |  |  |  |  |  |

| **Table 7.** Interactive Associations between Racial Discrimination and Religious Coping Predicting Diastolic Blood Pressure in African American Women, HANDLS Study (N = 410) |  |  |  |  |  |  |  |  |  |  |
| --- | --- | --- | --- | --- | --- | --- | --- | --- | --- | --- |
|  | Model 1 |  |  |  |  | Model 2 |  |  |  |  |
| *Variables* | *b* | SE | *t* | *p* | η^2^ | *b* | SE | *t* | *p* | η^2^ |
| Age | 0.09 | 0.06 | 1.41 | .159 | .010 | 0.08 | 0.06 | 1.37 | .171 | .010 |
| Socioeconomic status | -2.16* | 1.03 | -2.11 | .036 | .009 | -2.12* | 1.03 | -2.06 | .040 | .009 |
| Medication use | 2.15 | 1.12 | 1.92 | .056 | .008 | 2.14 | 1.12 | 1.91 | .057 | .008 |
| Racial discrimination | -0.14 | 1.02 | -0.14 | .887 | .000 | 0.06 | 1.03 | 0.06 | .952 | .000 |
| Religious coping | 0.14 | 0.55 | 0.26 | .794 | .000 | 0.80 | 0.83 | 0.97 | .334 | .000 |
| Racial discrimination × Religious coping |  |  |  |  |  | -1.16 | 1.10 | -1.06 | .291 | .003 |
| **Note**. * *p* < .05, ** *p* < .01, *** *p* < .001. **Abbreviations**. HANDLS Study = Healthy Aging in Neighborhoods of Diversity across the Life Span Study. SE = Standard error. Socioeconomic status = low, reference group. Medication use = Not currently using any antihypertensive, antidiabetic, or antilipidemic agent or medication. Racial discrimination = none, reference group. |  |  |  |  |  |  |  |  |  |  |

| **Table 8.** Interactive Associations between Racial Discrimination and Religious Coping Predicting Glycated Hemoglobin (HbA1c) in African American Women, HANDLS Study (N = 410) |  |  |  |  |  |  |  |  |  |  |
| --- | --- | --- | --- | --- | --- | --- | --- | --- | --- | --- |
|  | Model 1 |  |  |  |  | Model 2 |  |  |  |  |
| *Variables* | *b* | SE | *t* | *p* | η^2^ | *b* | SE | *t* | *p* | η^2^ |
| Age | 0.00** | 0.00 | 2.75 | .006 | .060 | 0.00** | 0.00 | 2.68 | .008 | .060 |
| Socioeconomic status | -0.03 | 0.02 | -1.73 | .084 | .004 | -0.03 | 0.02 | -1.64 | .101 | .004 |
| Medication use | 0.10*** | 0.02 | 5.51 | .000 | .070 | 0.10*** | 0.02 | 5.52 | .000 | .070 |
| Racial discrimination | 0.02 | 0.02 | 1.41 | .158 | .004 | 0.03 | 0.02 | 1.82 | .069 | .004 |
| Religious coping | 0.00 | 0.01 | 0.34 | .735 | .000 | 0.02 | 0.01 | 1.92 | .056 | .000 |
| Racial discrimination × Religious coping |  |  |  |  |  | -0.04* | 0.02 | -2.26 | .025 | .010 |
| **Note**. * *p* < .05, ** *p* < .01, *** *p* < .001. **Abbreviations**. HANDLS Study = Healthy Aging in Neighborhoods of Diversity across the Life Span Study. SE = Standard error. Socioeconomic status = low, reference group. Medication use = Not currently using any antihypertensive, antidiabetic, or antilipidemic agent or medication. Racial discrimination = none, reference group. |  |  |  |  |  |  |  |  |  |  |

| **Table 9.** Interactive Associations between Racial Discrimination and Religious Coping Predicting Body Mass Index in African American Women, HANDLS Study (N = 410) |  |  |  |  |  |  |  |  |  |  |
| --- | --- | --- | --- | --- | --- | --- | --- | --- | --- | --- |
|  | Model 1 |  |  |  |  | Model 2 |  |  |  |  |
| *Variables* | *b* | SE | *t* | *p* | η^2^ | *b* | SE | *t* | *p* | η^2^ |
| Age | 0.00* | 0.00 | -2.09 | .037 | .002 | 0.00* | 0.00 | -2.17 | .031 | .002 |
| Socioeconomic status | 0.03 | 0.02 | 1.30 | .195 | .006 | 0.03 | 0.02 | 1.39 | .167 | .006 |
| Medication use | 0.19*** | 0.03 | 7.02 | .000 | .100 | 0.19*** | 0.03 | 7.03 | .000 | .100 |
| Racial discrimination | -0.01 | 0.02 | -0.32 | .749 | .000 | 0.00 | 0.02 | 0.06 | .954 | .000 |
| Religious coping | 0.00 | 0.01 | -0.37 | .710 | .000 | 0.02 | 0.02 | 1.24 | .216 | .000 |
| Racial discrimination × Religious coping |  |  |  |  |  | -0.05* | 0.03 | -1.98 | .048 | .009 |
| **Note**. * *p* < .05, ** *p* < .01, *** *p* < .001. **Abbreviations**. HANDLS Study = Healthy Aging in Neighborhoods of Diversity across the Life Span Study. SE = Standard error. Socioeconomic status = low, reference group. Medication use = Not currently using any antihypertensive, antidiabetic, or antilipidemic agent or medication. Racial discrimination = none, reference group. |  |  |  |  |  |  |  |  |  |  |

| **Table 10.** Interactive Associations between Racial Discrimination and Religious Coping Predicting Cholesterol in African American Women, HANDLS Study (N = 410) |  |  |  |  |  |  |  |  |  |  |
| --- | --- | --- | --- | --- | --- | --- | --- | --- | --- | --- |
|  | Model 1 |  |  |  |  | Model 2 |  |  |  |  |
| *Variables* | *b* | SE | *t* | *p* | η^2^ | *b* | SE | *t* | *p* | η^2^ |
| Age | 0.39 | 0.21 | 1.85 | .065 | .020 | 0.40 | 0.21 | 1.86 | .064 | .020 |
| Socioeconomic status | 5.85 | 3.60 | 1.62 | .105 | .008 | 5.79 | 3.61 | 1.60 | .109 | .008 |
| Medication use | 7.21 | 3.94 | 1.83 | .067 | .008 | 7.23 | 3.94 | 1.83 | .067 | .008 |
| Racial discrimination | -5.55 | 3.56 | -1.56 | .120 | .006 | -5.82 | 3.63 | -1.60 | .110 | .006 |
| Religious coping | 3.12 | 1.93 | 1.62 | .106 | .006 | 2.27 | 2.92 | 0.78 | .436 | .006 |
| Racial discrimination × Religious coping |  |  |  |  |  | 1.48 | 3.85 | 0.39 | .700 | .000 |
| **Note**. * *p* < .05, ** *p* < .01, *** *p* < .001. **Abbreviations**. HANDLS Study = Healthy Aging in Neighborhoods of Diversity across the Life Span Study. SE = Standard error. Socioeconomic status = low, reference group. Medication use = Not currently using any antihypertensive, antidiabetic, or antilipidemic agent or medication. Racial discrimination = none, reference group. |  |  |  |  |  |  |  |  |  |  |

| **Table 11.** Interactive Associations between Racial Discrimination and Religious Coping Predicting Systolic Blood Pressure in African American Men, Sensitivity Testing: Depressive Symptoms |  |  |  |  |
| --- | --- | --- | --- | --- |
| *Variables* | *b* | SE | *t* | *p* |
| Depressive symptoms | 0.08 | 0.08 | 0.97 | .334 |
| Age | 0.32** | 0.09 | 3.51 | .001 |
| Socioeconomic status | 0.75 | 1.61 | 0.47 | .641 |
| Medication use | 4.75** | 1.74 | 2.73 | .007 |
| Racial discrimination | 2.78 | 1.70 | 1.64 | .102 |
| Religious coping | -0.81 | 0.96 | -0.84 | .400 |
| Racial discrimination × Religious coping | 4.34** | 1.55 | 2.81 | .005 |
| **Note**. * *p* < .05, ** *p* < .01, *** *p* < .001. **Abbreviations**. HANDLS Study = Healthy Aging in Neighborhoods of Diversity across the Life Span Study. SE = Standard error. Socioeconomic status = low, reference group. Medication use = Not currently using any antihypertensive, antidiabetic, or antilipidemic agent or medication. Racial discrimination = none, reference group. |  |  |  |  |

| **Table 12.** Interactive Associations between Racial Discrimination and Religious Coping Predicting Systolic Blood Pressure in African American Men, Sensitivity Testing: Biobehavioral Factors / Substance Use |  |  |  |  |
| --- | --- | --- | --- | --- |
| *Variables* | *b* | SE | *t* | *p* |
| Cigarette user status | 0.17 | 2.07 | 0.08 | .935 |
| Alcohol drinker status | 0.23 | 2.62 | 0.09 | .931 |
| Illicit drug use | 0.49 | 1.85 | 0.27 | .791 |
| Substance use coping | 0.49 | 0.42 | 1.19 | .237 |
| Age | 0.31** | 0.09 | 3.34 | .001 |
| Socioeconomic status | 0.78 | 1.64 | 0.47 | .635 |
| Medication use | 5.18** | 1.78 | 2.91 | .004 |
| Racial discrimination | 2.83 | 1.71 | 1.66 | .098 |
| Religious coping | -0.74 | 0.97 | -0.76 | .445 |
| Racial discrimination × Religious coping | 4.39** | 1.56 | 2.82 | .005 |
| **Note**. * *p* < .05, ** *p* < .01, *** *p* < .001. **Abbreviations**. HANDLS Study = Healthy Aging in Neighborhoods of Diversity across the Life Span Study. SE = Standard error. Socioeconomic status = low, reference group. Medication use = Not currently using any antihypertensive, antidiabetic, or antilipidemic agent or medication. Racial discrimination = none, reference group. Cigarette use = never used / have not used > 6 months, reference group. Alcohol use = never used / have not used > 6 months, reference group. Illicit drug use = has used (marijuana, opiates, or cocaine/crack) at least once within < 6 months, reference group.  Substance use coping = Z-score (mean-cetnered) taken for the subscale, Brief COPE Inventory (Carver, 1997). |  |  |  |  |

| **Table 13.** Interactive Associations between Racial Discrimination and Religious Coping Predicting Systolic Blood Pressure in African American Men, Sensitivity Testing: Social Support Factors |  |  |  |  |
| --- | --- | --- | --- | --- |
| *Variables* | *b* | SE | *t* | *p* |
| Marital status | 0.08 | 1.59 | 0.05 | .961 |
| Instrumental social support coping | 0.54 | 0.58 | 0.93 | .351 |
| Emotional social support coping | 0.05 | 0.59 | 0.08 | .935 |
| Age | 0.32** | 0.09 | 3.48 | .001 |
| Socioeconomic status | 0.40 | 1.59 | 0.25 | .802 |
| Medication use | 4.86** | 1.75 | 2.78 | .006 |
| Racial discrimination | 2.76 | 1.70 | 1.62 | .106 |
| Religious coping | -1.21 | 1.00 | -1.21 | .227 |
| Racial discrimination × Religious coping | 4.32** | 1.56 | 2.78 | .006 |
| **Note**. * *p* < .05, ** *p* < .01, *** *p* < .001. **Abbreviations**. HANDLS Study = Healthy Aging in Neighborhoods of Diversity across the Life Span Study. SE = Standard error. Socioeconomic status = low, reference group. Medication use = Not currently using any antihypertensive, antidiabetic, or antilipidemic agent or medication. Racial discrimination = none, reference group. Marital status = married/partnered, reference group. Instrumental coping = instrumental social support coping use, Z-score (mean-centered) taken for the subscale, Brief COPE Inventory (Carver, 1997). Emotional coping = emotional social support coping use, Z-score (mean-centered) taken for the subscale, Brief COPE Inventory (Carver, 1997). |  |  |  |  |

| **Table 14.** Interactive Associations between Racial Discrimination and Religious Coping Predicting Systolic Blood Pressure in African American Men, Sensitivity Testing: Biomedical Factors |  |  |  |  |
| --- | --- | --- | --- | --- |
| *Variables* | *b* | SE | *t* | *p* |
| Medical history of CVDs | 0.52 | 2.12 | 0.24 | .808 |
| Health insurance status | -2.52 | 1.76 | -1.43 | .155 |
| Age | 0.35*** | 0.10 | 3.63 | .000 |
| Socioeconomic status | 0.89 | 1.62 | 0.55 | .581 |
| Medication use | 5.08** | 1.78 | 2.86 | .005 |
| Racial discrimination | 3.16 | 1.73 | 1.83 | .068 |
| Religious coping | -0.87 | 0.96 | -0.91 | .364 |
| Racial discrimination × Religious coping | 4.15** | 1.56 | 2.67 | .008 |
| **Note**. * *p* < .05, ** *p* < .01, *** *p* < .001. **Abbreviations**. HANDLS Study = Healthy Aging in Neighborhoods of Diversity across the Life Span Study. SE = Standard error. Socioeconomic status = low, reference group. Medication use = Not currently using any antihypertensive, antidiabetic, or antilipidemic agent or medication. Racial discrimination = none, reference group. Prior CVD(s) = No medical history of prior CVD(s), reference group. Health insurance = uninsured, reference group. |  |  |  |  |

| **Table 15.** Interactive Associations between Racial Discrimination and Religious Coping Predicting Systolic Blood Pressure in African American Men, Sensitivity Testing: Body Mass Index |  |  |  |  |
| --- | --- | --- | --- | --- |
| *Variables* | *b* | SE | *t* | *p* |
| BMI | 0.45** | 0.13 | 3.37 | .001 |
| Age | 0.35*** | 0.09 | 3.87 | .000 |
| Socioeconomic status | -0.57 | 1.57 | -0.36 | .719 |
| Medication use | 3.13 | 1.79 | 1.75 | .081 |
| Racial discrimination | 2.16 | 1.68 | 1.29 | .199 |
| Religious coping | -0.95 | 0.95 | -1.00 | .317 |
| Racial discrimination × Religious coping | 3.98* | 1.53 | 2.61 | .010 |
| **Note**. * *p* < .05, ** *p* < .01, *** *p* < .001. **Abbreviations**. HANDLS Study = Healthy Aging in Neighborhoods of Diversity across the Life Span Study. SE = Standard error. Socioeconomic status = low, reference group. Medication use = Not currently using any antihypertensive, antidiabetic, or antilipidemic agent or medication. Racial discrimination = none, reference group. |  |  |  |  |

| **Table 16.** Interactive Associations between Racial Discrimination and Religious Coping Predicting Diastolic Blood Pressure in African American Men, Sensitivity Testing: Depressive Symptoms |  |  |  |  |
| --- | --- | --- | --- | --- |
| *Variables* | *b* | SE | *t* | *p* |
| Depressive symptoms | 0.06 | 0.06 | 1.07 | .288 |
| Age | -0.01 | 0.07 | -0.08 | .939 |
| Socioeconomic status | 1.44 | 1.19 | 1.21 | .228 |
| Medication use | 2.98* | 1.29 | 2.30 | .022 |
| Racial discrimination | 2.78* | 1.26 | 2.21 | .028 |
| Religious coping | 0.66 | 0.72 | 0.92 | .356 |
| Racial discrimination × Religious coping | 2.77* | 1.15 | 2.41 | .017 |
| **Note**. * *p* < .05, ** *p* < .01, *** *p* < .001. **Abbreviations**. HANDLS Study = Healthy Aging in Neighborhoods of Diversity across the Life Span Study. SE = Standard error. Socioeconomic status = low, reference group. Medication use = Not currently using any antihypertensive, antidiabetic, or antilipidemic agent or medication. Racial discrimination = none, reference group. |  |  |  |  |

| **Table 17.** Interactive Associations between Racial Discrimination and Religious Coping Predicting Diastolic Blood Pressure in African American Men, Sensitivity Testing: Biobehavioral Factors / Substance Use |  |  |  |  |
| --- | --- | --- | --- | --- |
| *Variables* | *b* | SE | *t* | *p* |
| Cigarette use | 0.81 | 1.54 | 0.53 | .599 |
| Alcohol use | -0.32 | 1.94 | -0.16 | .871 |
| Illicit drug use | 0.17 | 1.38 | 0.12 | .905 |
| Substance use coping | 0.22 | 0.31 | 0.72 | .474 |
| Age | -0.01 | 0.07 | -0.19 | .850 |
| Socioeconomic status | 1.48 | 1.22 | 1.21 | .227 |
| Medication use | 3.25* | 1.33 | 2.45 | .015 |
| Racial discrimination | 2.78* | 1.27 | 2.19 | .029 |
| Religious coping | 0.68 | 0.72 | 0.93 | .351 |
| Racial discrimination × Religious coping | 2.78* | 1.16 | 2.40 | .017 |
| **Note**. * *p* < .05, ** *p* < .01, *** *p* < .001. **Abbreviations**. HANDLS Study = Healthy Aging in Neighborhoods of Diversity across the Life Span Study. SE = Standard error. Socioeconomic status = low, reference group. Medication use = Not currently using any antihypertensive, antidiabetic, or antilipidemic agent or medication. Racial discrimination = none, reference group. Cigarette use = never used / have not used > 6 months, reference group. Alcohol use = never used / have not used > 6 months, reference group. Illicit drug use = has used (marijuana, opiates, or cocaine/crack) at least once within < 6 months, reference group.  Substance use coping = Z-score (mean-centered) taken for the subscale, Brief COPE Inventory (Carver, 1997). |  |  |  |  |

| **Table 18.** Interactive Associations between Racial Discrimination and Religious Coping Predicting Diastolic Blood Pressure in African American Men, Sensitivity Testing: Social Support Factors |  |  |  |  |
| --- | --- | --- | --- | --- |
| *Variables* | *b* | SE | *t* | *p* |
| Marital status | -0.26 | 1.18 | -0.22 | .828 |
| Instrumental social support coping | 0.46 | 0.43 | 1.07 | .284 |
| Emotional social support coping | 0.13 | 0.43 | 0.30 | .768 |
| Age | 0.00 | 0.07 | -0.06 | .950 |
| Socioeconomic status | 1.21 | 1.18 | 1.03 | .305 |
| Medication use | 3.08* | 1.30 | 2.37 | .018 |
| Racial discrimination | 2.79* | 1.26 | 2.21 | .028 |
| Religious coping | 0.29 | 0.74 | 0.39 | .693 |
| Racial discrimination × Religious coping | 2.75* | 1.15 | 2.38 | .018 |
| **Note**. * *p* < .05, ** *p* < .01, *** *p* < .001. **Abbreviations**. HANDLS Study = Healthy Aging in Neighborhoods of Diversity across the Life Span Study. SE = Standard error. Socioeconomic status = low, reference group. Medication use = Not currently using any antihypertensive, antidiabetic, or antilipidemic agent or medication. Racial discrimination = none, reference group. Marital status = married/partnered, reference group. Instrumental coping = instrumental social support coping use, Z-score (mean-centered) taken for the subscale, Brief COPE Inventory (Carver, 1997). Emotional coping = emotional social support coping use, Z-score (mean-centered) taken for the subscale, Brief COPE Inventory (Carver, 1997). |  |  |  |  |

| **Table 19.** Interactive Associations between Racial Discrimination and Religious Coping Predicting Diastolic Blood Pressure in African American Men, Sensitivity Testing: Biomedical Factors |  |  |  |  |
| --- | --- | --- | --- | --- |
| *Variables* | *b* | SE | *t* | *p* |
| Medical history of CVDs | -0.30 | 1.58 | -0.19 | .852 |
| Health insurance status | 0.12 | 1.31 | 0.09 | .926 |
| Age | -0.01 | 0.07 | -0.11 | .909 |
| Socioeconomic status | 1.10 | 1.20 | 0.92 | .360 |
| Medication use | 3.04* | 1.32 | 2.30 | .022 |
| Racial discrimination | 2.67* | 1.28 | 2.08 | .038 |
| Religious coping | 0.59 | 0.71 | 0.83 | .409 |
| Racial discrimination × Religious coping | 2.82* | 1.16 | 2.44 | .015 |
| **Note**. * *p* < .05, ** *p* < .01, *** *p* < .001. **Abbreviations**. HANDLS Study = Healthy Aging in Neighborhoods of Diversity across the Life Span Study. SE = Standard error. Socioeconomic status = low, reference group. Medication use = Not currently using any antihypertensive, antidiabetic, or antilipidemic agent or medication. Racial discrimination = none, reference group. Prior CVD(s) = No medical history of prior CVD(s), reference group. Health insurance = uninsured, reference group. |  |  |  |  |

| **Table 20.** Interactive Associations between Racial Discrimination and Religious Coping Predicting Diastolic Blood Pressure in African American Men, Sensitivity Testing: Body Mass Index |  |  |  |  |
| --- | --- | --- | --- | --- |
| *Variables* | *b* | SE | *t* | *p* |
| BMI | 0.17 | 0.10 | 1.66 | .098 |
| Age | 0.00 | 0.07 | 0.06 | .952 |
| Socioeconomic status | 0.80 | 1.18 | 0.68 | .499 |
| Medication use | 2.40 | 1.34 | 1.78 | .075 |
| Racial discrimination | 2.51* | 1.26 | 1.99 | .048 |
| Religious coping | 0.57 | 0.71 | 0.81 | .419 |
| Racial discrimination × Religious coping | 2.65* | 1.15 | 2.31 | .022 |
| **Note**. * *p* < .05, ** *p* < .01, *** *p* < .001. **Abbreviations**. HANDLS Study = Healthy Aging in Neighborhoods of Diversity across the Life Span Study. SE = Standard error. Socioeconomic status = low, reference group. Medication use = Not currently using any antihypertensive, antidiabetic, or antilipidemic agent or medication. Racial discrimination = none, reference group. |  |  |  |  |

| **Table 21.** Interactive Associations between Racial Discrimination and Religious Coping Predicting Glycated Hemoglobin (HbA1c) in African American Men, Sensitivity Testing: Depressive Symptoms |  |  |  |  |
| --- | --- | --- | --- | --- |
| *Variables* | *b* | SE | *t* | *p* |
| Depressive symptoms | 0.000 | 0.001 | 0.001 | .999 |
| Age | -0.001 | 0.001 | -0.551 | .582 |
| Socioeconomic status | 0.020 | 0.018 | 1.141 | .255 |
| Medication use | 0.157*** | 0.019 | 8.206 | .000 |
| Racial discrimination | 0.008 | 0.019 | 0.440 | .660 |
| Religious coping | -0.005 | 0.011 | -0.444 | .657 |
| Racial discrimination × Religious coping | 0.038* | 0.017 | 2.210 | .028 |
| **Note**. * *p* < .05, ** *p* < .01, *** *p* < .001. **Abbreviations**. HANDLS Study = Healthy Aging in Neighborhoods of Diversity across the Life Span Study. SE = Standard error. Socioeconomic status = low, reference group. Medication use = Not currently using any antihypertensive, antidiabetic, or antilipidemic agent or medication. Racial discrimination = none, reference group. |  |  |  |  |

| **Table 22.** Interactive Associations between Racial Discrimination and Religious Coping Predicting Glycated Hemoglobin (HbA1c) in African American Men, Sensitivity Testing: Biobehavioral Factors / Substance Use |  |  |  |  |
| --- | --- | --- | --- | --- |
| *Variables* | *b* | SE | *t* | *p* |
| Cigarette use | -0.035 | 0.022 | -1.572 | .117 |
| Alcohol use | -0.025 | 0.028 | -0.879 | .380 |
| Illicit drug use | -0.002 | 0.020 | -0.124 | .901 |
| Substance use coping | -0.009* | 0.005 | -2.056 | .041 |
| Age | 0.000 | 0.001 | -0.215 | .830 |
| Socioeconomic status | 0.006 | 0.018 | 0.349 | .728 |
| Medication use | 0.146* | 0.019 | 7.557 | .000 |
| Racial discrimination | 0.004 | 0.018 | 0.200 | .842 |
| Religious coping | -0.007 | 0.011 | -0.666 | .506 |
| Racial discrimination × Religious coping | 0.036* | 0.017 | 2.157 | .032 |
| **Note**. * *p* < .05, ** *p* < .01, *** *p* < .001. **Abbreviations**. HANDLS Study = Healthy Aging in Neighborhoods of Diversity across the Life Span Study. SE = Standard error. Socioeconomic status = low, reference group. Medication use = Not currently using any antihypertensive, antidiabetic, or antilipidemic agent or medication. Racial discrimination = none, reference group. Cigarette use = never used / have not used > 6 months, reference group. Alcohol use = never used / have not used > 6 months, reference group. Illicit drug use = has used (marijuana, opiates, or cocaine/crack) at least once within < 6 months, reference group.  Substance use coping = Z-score (mean-centered)  taken for the subscale, Brief COPE Inventory (Carver, 1997). |  |  |  |  |

| **Table 23.** Interactive Associations between Racial Discrimination and Religious Coping Predicting Glycated Hemoglobin (HbA1c) in African American Men, Sensitivity Testing: Social Support Factors |  |  |  |  |
| --- | --- | --- | --- | --- |
| *Variables* | *b* | SE | *t* | *p* |
| Marital status | -0.009 | 0.017 | -0.516 | .606 |
| Instrumental social support coping | 0.002 | 0.006 | 0.296 | .767 |
| Emotional social support coping | -0.003 | 0.006 | -0.389 | .697 |
| Age | -0.001 | 0.001 | -0.633 | .527 |
| Socioeconomic status | 0.021 | 0.017 | 1.201 | .230 |
| Medication use | 0.157*** | 0.019 | 8.206 | .000 |
| Racial discrimination | 0.009 | 0.019 | 0.468 | .640 |
| Religious coping | -0.005 | 0.011 | -0.413 | .680 |
| Racial discrimination × Religious coping | 0.039* | 0.017 | 2.258 | .025 |
| **Note**. * *p* < .05, ** *p* < .01, *** *p* < .001. **Abbreviations**. HANDLS Study = Healthy Aging in Neighborhoods of Diversity across the Life Span Study. SE = Standard error. Socioeconomic status = low, reference group. Medication use = Not currently using any antihypertensive, antidiabetic, or antilipidemic agent or medication. Racial discrimination = none, reference group. Marital status = married/partnered, reference group. Instrumental coping = instrumental social support coping use, Z-score (mean-centered) taken for the subscale, Brief COPE Inventory (Carver, 1997). Emotional coping = emotional social support coping use, Z-score (mean-centered) taken for the subscale, Brief COPE Inventory (Carver, 1997). |  |  |  |  |

| **Table 24.** Interactive Associations between Racial Discrimination and Religious Coping Predicting Glycated Hemoglobin (HbA1c) in African American Men, Sensitivity Testing: Biomedical Factors |  |  |  |  |
| --- | --- | --- | --- | --- |
| *Variables* | *b* | SE | *t* | *p* |
| Medical history of CVDs | 0.000 | 0.023 | -0.010 | .992 |
| Health insurance status | 0.011 | 0.019 | 0.555 | .579 |
| Age | -0.001 | 0.001 | -0.674 | .501 |
| Socioeconomic status | 0.018 | 0.018 | 1.028 | .305 |
| Medication use | 0.156*** | 0.020 | 7.967 | .000 |
| Racial discrimination | 0.006 | 0.019 | 0.329 | .742 |
| Religious coping | -0.005 | 0.011 | -0.452 | .652 |
| Racial discrimination × Religious coping | 0.038* | 0.017 | 2.252 | .025 |
| **Note**. * *p* < .05, ** *p* < .01, *** *p* < .001. **Abbreviations**. HANDLS Study = Healthy Aging in Neighborhoods of Diversity across the Life Span Study. SE = Standard error. Socioeconomic status = low, reference group. Medication use = Not currently using any antihypertensive, antidiabetic, or antilipidemic agent or medication. Racial discrimination = none, reference group. Prior CVD(s) = No medical history of prior CVD(s), reference group. Health insurance = uninsured, reference group. |  |  |  |  |

| **Table 25.** Interactive Associations between Racial Discrimination and Religious Coping Predicting Glycated Hemoglobin (HbA1c) in African American Men, Sensitivity Testing: Body Mass Index |  |  |  |  |
| --- | --- | --- | --- | --- |
| *Variables* | *b* | SE | *t* | *p* |
| BMI | 0.008*** | 0.001 | 5.545 | .000 |
| Age | 0.000 | 0.001 | 0.061 | .952 |
| Socioeconomic status | 0.003 | 0.017 | 0.187 | .851 |
| Medication use | 0.128*** | 0.019 | 6.682 | .000 |
| Racial discrimination | -0.001 | 0.018 | -0.061 | .951 |
| Religious coping | -0.006 | 0.010 | -0.560 | .576 |
| Racial discrimination × Religious coping | 0.030 | 0.016 | 1.863 | .063 |
| **Note**. * *p* < .05, ** *p* < .01, *** *p* < .001. **Abbreviations**. HANDLS Study = Healthy Aging in Neighborhoods of Diversity across the Life Span Study. SE = Standard error. Socioeconomic status = low, reference group. Medication use = Not currently using any antihypertensive, antidiabetic, or antilipidemic agent or medication. Racial discrimination = none, reference group. |  |  |  |  |

| **Table 26.** Interactive Associations between Racial Discrimination and Religious Coping Predicting Glycated Hemoglobin (HbA1c) in African American Women, Sensitivity Testing: Depressive Symptoms |  |  |  |  |
| --- | --- | --- | --- | --- |
| *Variables* | *b* | SE | *t* | *p* |
| Depressive symptoms | 0.000 | 0.001 | 0.439 | .661 |
| Age | 0.003** | 0.001 | 2.711 | .007 |
| Socioeconomic status | -0.024 | 0.016 | -1.488 | .137 |
| Medication use | 0.096*** | 0.017 | 5.503 | .000 |
| Racial discrimination | 0.031 | 0.016 | 1.868 | .062 |
| Religious coping | 0.025 | 0.013 | 1.925 | .055 |
| Racial discrimination × Religious coping | -0.038* | 0.017 | -2.227 | .026 |
| **Note**. * *p* < .05, ** *p* < .01, *** *p* < .001. **Abbreviations**. HANDLS Study = Healthy Aging in Neighborhoods of Diversity across the Life Span Study. SE = Standard error. Socioeconomic status = low, reference group. Medication use = Not currently using any antihypertensive, antidiabetic, or antilipidemic agent or medication. Racial discrimination = none, reference group. |  |  |  |  |

| **Table 27.** Interactive Associations between Racial Discrimination and Religious Coping Predicting Glycated Hemoglobin (HbA1c) in African American Women, Sensitivity Testing: Biobehavioral Factors / Substance Use |  |  |  |  |
| --- | --- | --- | --- | --- |
| *Variables* | *b* | SE | *t* | *p* |
| Cigarette use | 0.022 | 0.018 | 1.241 | .215 |
| Alcohol use | -0.022 | 0.019 | -1.166 | .244 |
| Illicit drug use | -0.012 | 0.018 | -0.677 | .499 |
| Substance use coping | -0.010* | 0.005 | -2.005 | .046 |
| Age | 0.002 | 0.001 | 2.446 | .015 |
| Socioeconomic status | -0.023 | 0.016 | -1.427 | .154 |
| Medication use | 0.094*** | 0.018 | 5.355 | .000 |
| Racial discrimination | 0.027 | 0.016 | 1.661 | .097 |
| Religious coping | 0.023 | 0.013 | 1.734 | .084 |
| Racial discrimination × Religious coping | -0.037* | 0.017 | -2.174 | .030 |
| **Note**. * *p* < .05, ** *p* < .01, *** *p* < .001. **Abbreviations**. HANDLS Study = Healthy Aging in Neighborhoods of Diversity across the Life Span Study. SE = Standard error. Socioeconomic status = low, reference group. Medication use = Not currently using any antihypertensive, antidiabetic, or antilipidemic agent or medication. Racial discrimination = none, reference group. Cigarette use = never used / have not used > 6 months, reference group. Alcohol use = never used / have not used > 6 months, reference group. Illicit drug use = has used (marijuana, opiates, or cocaine/crack) at least once within < 6 months, reference group.  Substance use coping = Z-score (mean-centered) taken for the subscale, Brief COPE Inventory (Carver, 1997). |  |  |  |  |

| **Table 28.** Interactive Associations between Racial Discrimination and Religious Coping Predicting Glycated Hemoglobin (HbA1c) in African American Women, Sensitivity Testing: Social Support Factors |  |  |  |  |
| --- | --- | --- | --- | --- |
| *Variables* | *b* | SE | *t* | *p* |
| Marital status | -0.001 | 0.017 | -0.062 | .951 |
| Instrumental social support coping | -0.005 | 0.006 | -0.863 | .389 |
| Emotional social support coping | 0.009 | 0.006 | 1.394 | .164 |
| Age | 0.003** | 0.001 | 2.741 | .006 |
| Socioeconomic status | -0.028 | 0.016 | -1.732 | .084 |
| Medication use | 0.096*** | 0.017 | 5.475 | .000 |
| Racial discrimination | 0.029 | 0.016 | 1.808 | .071 |
| Religious coping | 0.024 | 0.013 | 1.802 | .072 |
| Racial discrimination × Religious coping | -0.039* | 0.017 | -2.269 | .024 |
| **Note**. * *p* < .05, ** *p* < .01, *** *p* < .001. **Abbreviations**. HANDLS Study = Healthy Aging in Neighborhoods of Diversity across the Life Span Study. SE = Standard error. Socioeconomic status = low, reference group. Medication use = Not currently using any antihypertensive, antidiabetic, or antilipidemic agent or medication. Racial discrimination = none, reference group. Marital status = married/partnered, reference group. Instrumental coping = instrumental social support coping use, Z-score (mean-centered) taken for the subscale, Brief COPE Inventory (Carver, 1997). Emotional coping = emotional social support coping use, Z-score (mean-centered) taken for the subscale, Brief COPE Inventory (Carver, 1997). |  |  |  |  |

| **Table 29.** Interactive Associations between Racial Discrimination and Religious Coping Predicting Glycated Hemoglobin (HbA1c) in African American Women, Sensitivity Testing: Biomedical Factors |  |  |  |  |
| --- | --- | --- | --- | --- |
| *Variables* | *b* | SE | *t* | *p* |
| Medical history of CVDs | -0.002 | 0.020 | -0.120 | .905 |
| Health insurance status | 0.035* | 0.017 | 1.991 | .047 |
| Age | 0.003** | 0.001 | 2.654 | .008 |
| Socioeconomic status | -0.034* | 0.017 | -2.064 | .040 |
| Medication use | 0.091*** | 0.018 | 5.096 | .000 |
| Racial discrimination | 0.028 | 0.016 | 1.748 | .081 |
| Religious coping | 0.024 | 0.013 | 1.900 | .058 |
| Racial discrimination × Religious coping | -0.039* | 0.017 | -2.291 | .022 |
| **Note**. * *p* < .05, ** *p* < .01, *** *p* < .001. **Abbreviations**. HANDLS Study = Healthy Aging in Neighborhoods of Diversity across the Life Span Study. SE = Standard error. Socioeconomic status = low, reference group. Medication use = Not currently using any antihypertensive, antidiabetic, or antilipidemic agent or medication. Racial discrimination = none, reference group. Prior CVD(s) = No medical history of prior CVD(s), reference group. Health insurance = uninsured, reference group. |  |  |  |  |

| **Table 30.** Interactive Associations between Racial Discrimination and Religious Coping Predicting Glycated Hemoglobin (HbA1c) in African American Women, Sensitivity Testing: Body Mass Index |  |  |  |  |
| --- | --- | --- | --- | --- |
| *Variables* | *b* | SE | *t* | *p* |
| BMI | 0.003*** | 0.001 | 3.519 | .000 |
| Age | 0.003** | 0.001 | 3.036 | .003 |
| Socioeconomic status | -0.029 | 0.016 | -1.822 | .069 |
| Medication use | 0.076*** | 0.018 | 4.209 | .000 |
| Racial discrimination | 0.029 | 0.016 | 1.823 | .069 |
| Religious coping | 0.022 | 0.013 | 1.704 | .089 |
| Racial discrimination × Religious coping | -0.033 | 0.017 | -1.930 | .054 |
| **Note**. * *p* < .05, ** *p* < .01, *** *p* < .001. **Abbreviations**. HANDLS Study = Healthy Aging in Neighborhoods of Diversity across the Life Span Study. SE = Standard error. Socioeconomic status = low, reference group. Medication use = Not currently using any antihypertensive, antidiabetic, or antilipidemic agent or medication. Racial discrimination = none, reference group. |  |  |  |  |

| **Table 31.** Interactive Associations between Racial Discrimination and Religious Coping Predicting Body Mass Index in African American Women, Sensitivity Testing: Depressive Symptoms |  |  |  |  |
| --- | --- | --- | --- | --- |
| *Variables* | *b* | SE | *t* | *p* |
| Depressive symptoms | 0.000 | 0.001 | -0.042 | .967 |
| Age | -0.003* | 0.001 | -2.146 | .032 |
| Socioeconomic status | 0.033 | 0.025 | 1.334 | .183 |
| Medication use | 0.186*** | 0.026 | 7.024 | .000 |
| Racial discrimination | 0.001 | 0.025 | 0.049 | .961 |
| Religious coping | 0.024 | 0.020 | 1.235 | .217 |
| Racial discrimination × Religious coping | -0.051* | 0.026 | -1.976 | .049 |
| **Note**. * *p* < .05, ** *p* < .01, *** *p* < .001. **Abbreviations**. HANDLS Study = Healthy Aging in Neighborhoods of Diversity across the Life Span Study. SE = Standard error. Socioeconomic status = low, reference group. Medication use = Not currently using any antihypertensive, antidiabetic, or antilipidemic agent or medication. Racial discrimination = none, reference group. |  |  |  |  |

| **Table 32.** Interactive Associations between Racial Discrimination and Religious Coping Predicting Body Mass Index in African American Women, Sensitivity Testing: Biobehavioral Factors / Substance Use |  |  |  |  |
| --- | --- | --- | --- | --- |
| *Variables* | *b* | SE | *t* | *p* |
| Cigarette use | -0.054* | 0.027 | -2.004 | .046 |
| Alcohol use | -0.038 | 0.028 | -1.352 | .177 |
| Illicit drug use | -0.031 | 0.027 | -1.143 | .254 |
| Substance use coping | -0.030*** | 0.007 | -4.033 | .000 |
| Age | -0.003* | 0.001 | -2.232 | .026 |
| Socioeconomic status | 0.021 | 0.024 | 0.857 | .392 |
| Medication use | 0.173*** | 0.026 | 6.590 | .000 |
| Racial discrimination | -0.008 | 0.024 | -0.323 | .747 |
| Religious coping | 0.008 | 0.019 | 0.421 | .674 |
| Racial discrimination × Religious coping | -0.041 | 0.025 | -1.630 | .104 |
| **Note**. * *p* < .05, ** *p* < .01, *** *p* < .001. **Abbreviations**. HANDLS Study = Healthy Aging in Neighborhoods of Diversity across the Life Span Study. SE = Standard error. Socioeconomic status = low, reference group. Medication use = Not currently using any antihypertensive, antidiabetic, or antilipidemic agent or medication. Racial discrimination = none, reference group. Cigarette use = never used / have not used > 6 months, reference group. Alcohol use = never used / have not used > 6 months, reference group. Illicit drug use = has used (marijuana, opiates, or cocaine/crack) at least once within < 6 months, reference group.  Substance use coping = Z-score (mean-centered) taken for the subscale, Brief COPE Inventory (Carver, 1997). |  |  |  |  |

| **Table 33.** Interactive Associations between Racial Discrimination and Religious Coping Predicting Body Mass Index in African American Women, Sensitivity Testing: Social Support Factors |  |  |  |  |
| --- | --- | --- | --- | --- |
| *Variables* | *b* | SE | *t* | *p* |
| Marital status | -0.009 | 0.026 | -0.353 | .724 |
| Instrumental social support coping | -0.002 | 0.009 | -0.261 | .794 |
| Emotional social support coping | 0.003 | 0.009 | 0.335 | .738 |
| Age | -0.003* | 0.001 | -2.148 | .032 |
| Socioeconomic status | 0.034 | 0.025 | 1.383 | .167 |
| Medication use | 0.186*** | 0.027 | 6.994 | .000 |
| Racial discrimination | 0.002 | 0.025 | 0.063 | .950 |
| Religious coping | 0.024 | 0.020 | 1.186 | .236 |
| Racial discrimination × Religious coping | -0.050 | 0.026 | -1.935 | .054 |
| **Note**. * *p* < .05, ** *p* < .01, *** *p* < .001. **Abbreviations**. HANDLS Study = Healthy Aging in Neighborhoods of Diversity across the Life Span Study. SE = Standard error. Socioeconomic status = low, reference group. Medication use = Not currently using any antihypertensive, antidiabetic, or antilipidemic agent or medication. Racial discrimination = none, reference group. Marital status = married/partnered, reference group. Instrumental coping = instrumental social support coping use, Z-score (mean-centered) taken for the subscale, Brief COPE Inventory (Carver, 1997). Emotional coping = emotional social support coping use, Z-score (mean-centered) taken for the subscale, Brief COPE Inventory (Carver, 1997). |  |  |  |  |

| **Table 34.** Interactive Associations between Racial Discrimination and Religious Coping Predicting Body Mass Index in African American Women, Sensitivity Testing: Biomedical Factors |  |  |  |  |
| --- | --- | --- | --- | --- |
| *Variables* | *b* | SE | *t* | *p* |
| Prior CVD(s) | -0.009 | 0.030 | -0.297 | .766 |
| Health insurance | 0.023 | 0.027 | 0.880 | .379 |
| Age | -0.003* | 0.001 | -2.124 | .034 |
| Socioeconomic status | 0.027 | 0.025 | 1.071 | .285 |
| Medication use | 0.184*** | 0.027 | 6.747 | .000 |
| Racial discrimination | 0.001 | 0.024 | 0.035 | .972 |
| Religious coping | 0.024 | 0.020 | 1.223 | .222 |
| Racial discrimination × Religious coping | -0.051* | 0.026 | -1.984 | .048 |
| **Note**. * *p* < .05, ** *p* < .01, *** *p* < .001. **Abbreviations**. HANDLS Study = Healthy Aging in Neighborhoods of Diversity across the Life Span Study. SE = Standard error. CVD(s) = Cardiovascular disease(s). Prior CVD(s) = No medical history of prior CVD(s), reference group. Health insurance = uninsured, reference group. Socioeconomic status = low, reference group. Medication use = Not currently using any antihypertensive, antidiabetic, or antilipidemic agent or medication. Racial discrimination = none, reference group. |  |  |  |  |

| **Table 35.** Inferential Statistics from Multiple Linear Regression Models Estimating 3-Way Interactions for Racial Discrimination × Religious Coping × Sex with Systolic Blood Pressure, HANDLS Study (N = 815) |  |  |  |  |
| --- | --- | --- | --- | --- |
| *Variables* | *b* | SE | *t* | *p* |
| Age | 0.52*** | 0.07 | 7.95 | .000 |
| Socioeconomic status | -1.52 | 1.13 | -1.35 | .178 |
| Medication use | 4.96*** | 1.24 | 4.01 | .000 |
| Racial discrimination | 1.00 | 1.53 | 0.65 | .513 |
| Religious coping | 1.31 | 1.23 | 1.06 | .288 |
| Sex | 0.75 | 1.56 | 0.48 | .631 |
| Racial discrimination × Religious coping | -1.39 | 1.62 | -0.86 | .392 |
| Racial discrimination × Sex | 1.50 | 2.38 | 0.63 | .529 |
| Religious coping × Sex | -2.29 | 1.60 | -1.43 | .154 |
| Racial discrimination × Religious coping × Sex | 5.47* | 2.32 | 2.36 | .019 |
| **Note**. * *p* < .05, ** *p* < .01, *** *p* < .001. **Abbreviations**. HANDLS Study = Healthy Aging in Neighborhoods of Diversity across the Life Span Study. SE = Standard error. Socioeconomic status = low, reference group. Sex = women, reference group. Medication use = Not currently using any antihypertensive, antidiabetic, or antilipidemic agent or medication. Racial discrimination = none, reference group. |  |  |  |  |

| **Table 36.** Inferential Statistics from Multiple Linear Regression Models Estimating 3-Way Interactions for Racial Discrimination × Religious Coping × Sex with Diastolic Blood Pressure, HANDLS Study (N = 815) |  |  |  |  |
| --- | --- | --- | --- | --- |
| *Variables* | *b* | SE | *t* | *p* |
| Age | 0.04 | 0.04 | 0.94 | .349 |
| Socioeconomic status | -0.66 | 0.77 | -0.85 | .395 |
| Medication use | 2.50** | 0.85 | 2.96 | .003 |
| Racial discrimination | 0.00 | 1.05 | 0.00 | .998 |
| Religious coping | 0.77 | 0.84 | 0.91 | .362 |
| Sex | 1.89 | 1.07 | 1.77 | .077 |
| Racial discrimination × Religious coping | -1.25 | 1.11 | -1.12 | .261 |
| Racial discrimination × Sex | 2.64 | 1.62 | 1.62 | .105 |
| Religious coping × Sex | -0.22 | 1.10 | -0.20 | .843 |
| Racial discrimination × Religious coping × Sex | 4.01* | 1.58 | 2.53 | .012 |
| **Note**. * *p* < .05, ** *p* < .01, *** *p* < .001. **Abbreviations**. HANDLS Study = Healthy Aging in Neighborhoods of Diversity across the Life Span Study. SE = Standard error. Socioeconomic status = low, reference group. Sex = women, reference group. Medication use = Not currently using any antihypertensive, antidiabetic, or antilipidemic agent or medication. Racial discrimination = none, reference group. |  |  |  |  |

| **Table 37.** Inferential Statistics from Multiple Linear Regression Models Estimating 3-Way Interactions for Racial Discrimination × Religious Coping × Sex with Glycated Hemoglobin (HbA1c), HANDLS Study (N = 815) |  |  |  |  |
| --- | --- | --- | --- | --- |
| *Variables* | *b* | SE | *t* | *p* |
| Age | 0.001 | 0.001 | 1.570 | .117 |
| Socioeconomic status | -0.006 | 0.012 | -0.546 | .585 |
| Medication use | 0.123*** | 0.013 | 9.550 | .000 |
| Racial discrimination | 0.027 | 0.016 | 1.707 | .088 |
| Religious coping | 0.024 | 0.013 | 1.854 | .064 |
| Sex | 0.018 | 0.016 | 1.102 | .271 |
| Racial discrimination × Religious coping | -0.040* | 0.017 | -2.352 | .019 |
| Racial discrimination × Sex | -0.022 | 0.025 | -0.879 | .380 |
| Religious coping × Sex | -0.029 | 0.017 | -1.751 | .080 |
| Racial discrimination × Religious coping × Sex | 0.076** | 0.024 | 3.133 | .002 |
| **Note**. * *p* < .05, ** *p* < .01, *** *p* < .001. **Abbreviations**. HANDLS Study = Healthy Aging in Neighborhoods of Diversity across the Life Span Study. SE = Standard error. Socioeconomic status = low, reference group. Sex = women, reference group. Medication use = Not currently using any antihypertensive, antidiabetic, or antilipidemic agent or medication. Racial discrimination = none, reference group. |  |  |  |  |

| **Table 38.** Inferential Statistics from Multiple Linear Regression Models Estimating 3-Way Interactions for Racial Discrimination × Religious Coping × Sex with Body Mass Index, HANDLS Study (N = 815) |  |  |  |  |
| --- | --- | --- | --- | --- |
| *Variables* | *b* | SE | *t* | *p* |
| Age | -0.003** | 0.001 | -2.766 | .006 |
| Socioeconomic status | 0.055** | 0.016 | 3.345 | .001 |
| Medication use | 0.159*** | 0.018 | 8.847 | .000 |
| Racial discrimination | 0.002 | 0.022 | 0.092 | .927 |
| Religious coping | 0.025 | 0.018 | 1.389 | .165 |
| Sex | -0.128*** | 0.023 | -5.662 | .000 |
| Racial discrimination × Religious coping | -0.052* | 0.024 | -2.221 | .027 |
| Racial discrimination × Sex | 0.040 | 0.034 | 1.154 | .249 |
| Religious coping × Sex | -0.015 | 0.023 | -0.648 | .517 |
| Racial discrimination × Religious coping × Sex | 0.074* | 0.034 | 2.193 | .029 |
| **Note**. * *p* < .05, ** *p* < .01, *** *p* < .001. **Abbreviations**. HANDLS Study = Healthy Aging in Neighborhoods of Diversity across the Life Span Study. SE = Standard error. Socioeconomic status = low, reference group. Sex = women, reference group. Medication use = Not currently using any antihypertensive, antidiabetic, or antilipidemic agent or medication. Racial discrimination = none, reference group. |  |  |  |  |

| **Table 39.** Inferential Statistics from Multiple Linear Regression Models Estimating 3-Way Interactions for Racial Discrimination × Religious Coping × Sex with Cholesterol, HANDLS Study (N = 815) |  |  |  |  |
| --- | --- | --- | --- | --- |
| *Variables* | *b* | SE | *t* | *p* |
| Age | 0.20 | 0.17 | 1.15 | .249 |
| Socioeconomic status | 2.43 | 3.00 | 0.81 | .418 |
| Medication use | 2.73 | 3.29 | 0.83 | .408 |
| Racial discrimination | -5.84 | 4.08 | -1.43 | .152 |
| Religious coping | 3.01 | 3.27 | 0.92 | .358 |
| Sex | -10.61* | 4.16 | -2.55 | .011 |
| Racial discrimination × Religious coping | 1.36 | 4.32 | 0.31 | .753 |
| Racial discrimination × Sex | 15.21* | 6.31 | 2.41 | .016 |
| Religious coping × Sex | -6.76 | 4.26 | -1.59 | .113 |
| Racial discrimination × Religious coping × Sex | 9.26 | 6.16 | 1.50 | .133 |
| **Note**. * *p* < .05, ** *p* < .01, *** *p* < .001. **Abbreviations**. HANDLS Study = Healthy Aging in Neighborhoods of Diversity across the Life Span Study. SE = Standard error. Socioeconomic status = low, reference group. Sex = women, reference group. Medication use = Not currently using any antihypertensive, antidiabetic, or antilipidemic agent or medication. Racial discrimination = none, reference group. |  |  |  |  |
